# Therapeutic activation of IL-22-producing innate lymphoid cells enhances host defenses to *Clostridioides difficile* infection

**DOI:** 10.1016/j.celrep.2025.115438

**Published:** 2025-03-25

**Authors:** Kevin S. Mears, Joshua E. Denny, Jeffrey R. Maslanka, Nontokozo V. Mdluli, Ellie N. Hulit, Rina Matsuda, Emma E. Furth, Charlie G. Buffie, Michael C. Abt

**Affiliations:** 1 Department of Microbiology, Perelman School of Medicine, University of Pennsylvania, Philadelphia, PA, USA; 2 Department of Molecular Virology and Microbiology, Baylor College of Medicine, Houston, TX, USA; 3 Department of Pathology, University of Pennsylvania Medical Center, Philadelphia, PA, USA; 4 Laboratory of Genetically Encoded Small Molecules, The Rockefeller University, New York, NY, USA; 5 Institute for Immunology and Immune Health, Perelman School of Medicine, University of Pennsylvania, Philadelphia, PA, USA; 6 Lead contact

## Abstract

*Clostridioides difficile* causes debilitating colitis via secreted toxins that disrupt the intestinal barrier, and toxemia is associated with severe disease. Thus, therapies that fortify the intestinal barrier will reduce the severity of infection. Innate lymphoid cells (ILCs) are critical in the defense against acute *C. difficile* infection and represent a promising therapeutic target to limit disease. Here, we report that oral administration of the Toll-like receptor (TLR) 7 agonist R848 limits intestinal damage and protects mice from lethal *C. difficile* infection without impacting pathogen burden or altering the intestinal microbiome. R848 induced interleukin (IL)-22 secretion by ILCs, leading to STAT3 phosphorylation in the intestinal epithelium and increased stem cell proliferation. Genetic ablation of ILCs, IL-22, or epithelial-specific STAT3 abrogated R848-mediated protection. R848 reduced intestinal permeability following infection and limited systemic toxin dissemination. Combined, these data identify an immunostimulatory molecule that activates IL-22 production in ILCs to enhance host tissue defenses following *C. difficile* infection.

## In brief

Mears et al. report that the oral administration of a TLR7 agonist R848 protects mice against severe disease and limits intestinal damage following *C. difficile* infection. R848 indirectly stimulated ILC3s to produce IL-22, which signaled on intestinal epithelial cells to improve barrier function and limit systemic toxin dissemination.

## Graphical Abstract

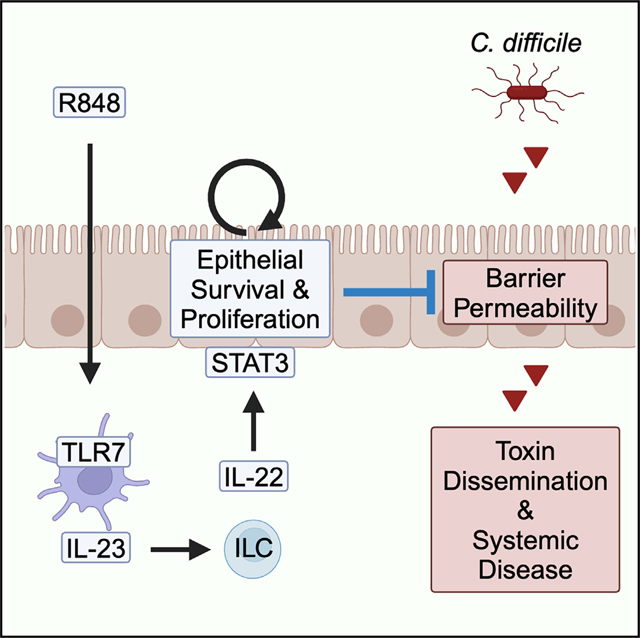

## INTRODUCTION

*Clostridioides difficile* is the most common cause of nosocomial gastrointestinal infections and is associated with significant morbidity and mortality worldwide.^1,2^ *C. difficile* infects the large intestine of susceptible individuals following disruption of the commensal microbiota, which is typically associated with broad-spectrum antibiotics use.^3^ Current antibiotic treatments are often ineffective, with approximately 20%–30% of patients experiencing recurrent infections.^1^ The pathogen’s main virulence factors are two potent secreted toxins, toxin A (TcdA) and toxin B (TcdB), that target epithelial cells leading to the breakdown of the intestinal barrier and robust inflammatory response.^4,5^ In addition to the intestinal pathology, systemic complications from severe *C. difficile* infection have been reported in patients, including cardiopulmonary arrest, acute respiratory distress syndrome, liver damage, and renal failure.^6–9^ Furthermore, the systemic dissemination of *C. difficile* toxin is associated with systemic manifestations of disease in animal models, and toxemia has been reported in patients in a few isolated cases.^10–12^ A recent report demonstrated that administering monoclonal antibodies against *C. difficile* toxin prevented extra-intestinal organ pathology without reducing intestinal damage, further supporting the role of circulating toxin in the development of systemic complications.^13^

The host immune response is an important determinant of disease outcome and comprises two distinct defense strategies: resistance and tolerance.^14–18^ While resistance mechanisms protect the host by reducing pathogen burden, disease tolerance reduces the negative fitness cost caused by the pathogen or the host immune response without directly affecting pathogen burden.^15–19^ Previous works have identified specific innate immune cell subsets and pathways that are critical in the defense against *C. difficile*. Type I and type III innate lymphoid cells (ILC1s and ILC3s) respond acutely during *C. difficile* infection by producing effector cytokines such as interferon (IFN)-γ, interleukin (IL)-22, and granulocyte-macrophage colony-stimulating factor (GM-CSF).^20,21^ These cytokines activate inflammatory cells, stimulate the production of antimicrobial factors, and promote repair mechanisms at the intestinal barrier.^22^ Importantly, mice deficient for ILCs exhibit greater mortality following *C. difficile* challenge,^20,21,23,24^ highlighting the importance of these cells in the defense against *C. difficile* infection and potential as targets for immune-based therapeutics. IL-22 is important in the defense against *C. difficile* infection by enhancing complement-mediated clearance of pathobionts in peripheral organs and regulating host glycosylation, which fuels the growth of commensal microbes that outcompete *C. difficile*.^25,26^ Mice deficient for IL-10 have elevated basal expression of IL-22, which reduces acute disease severity following *C. difficile* infection; however, sustained aberrant inflammation in the context of IL-10 deficiency leads to dysbiosis and increased susceptibility to colitis.^27–29^ Thus, identifying ways to transiently target IL-22 production may help mitigate the burden of *C. difficile* infection.

Commensal bacteria that reside in the intestines can activate basal immune defenses against enteric pathogens.^30,31^ Similarly, enteric viruses can prevent the outgrowth of pathogenic bacteria and regulate the inflammatory response.^32^ Resiquimod (R848) is a small-molecule immune response modifier that activates Toll-like receptor (TLR) 7, which detects viral single-stranded RNA genomes.^33^ R848 has antiviral and antitumor activity by stimulating cytokine production.^34–37^ It was previously reported that oral administration of R848 reduced colonization of vancomycin-resistant enterococci (VREs) in the small intestine.^38^ R848 signals through TLR7 on CD11c^+^ dendritic cells to induce IL-23 secretion, which further stimulates IL-22 production by ILCs.^38,39^

In this study, we sought to therapeutically enhance host defense mechanisms against *C. difficile* infection without collateral tissue damage. We demonstrate that R848 promotes host tolerance of acute disease following lethal *C. difficile* challenge without affecting pathogen colonization or toxin titers. Increased disease tolerance was mediated by large intestinal ILCs producing IL-22 that signaled on intestinal epithelial cells to activate the STAT3 pathway and enhance epithelial regeneration. R848 treatment reduced the damage to the intestinal barrier following infection and limited the systemic dissemination of *C. difficile* toxins, thereby preventing systemic disease. Our study demonstrates that transiently targeting IL-22 production from ILCs may be beneficial in reducing morbidity in *C. difficile*-infected patients.

## RESULTS

### R848 treatment protects mice against mortality following *C. diffizile* infection

**Figure 1.**
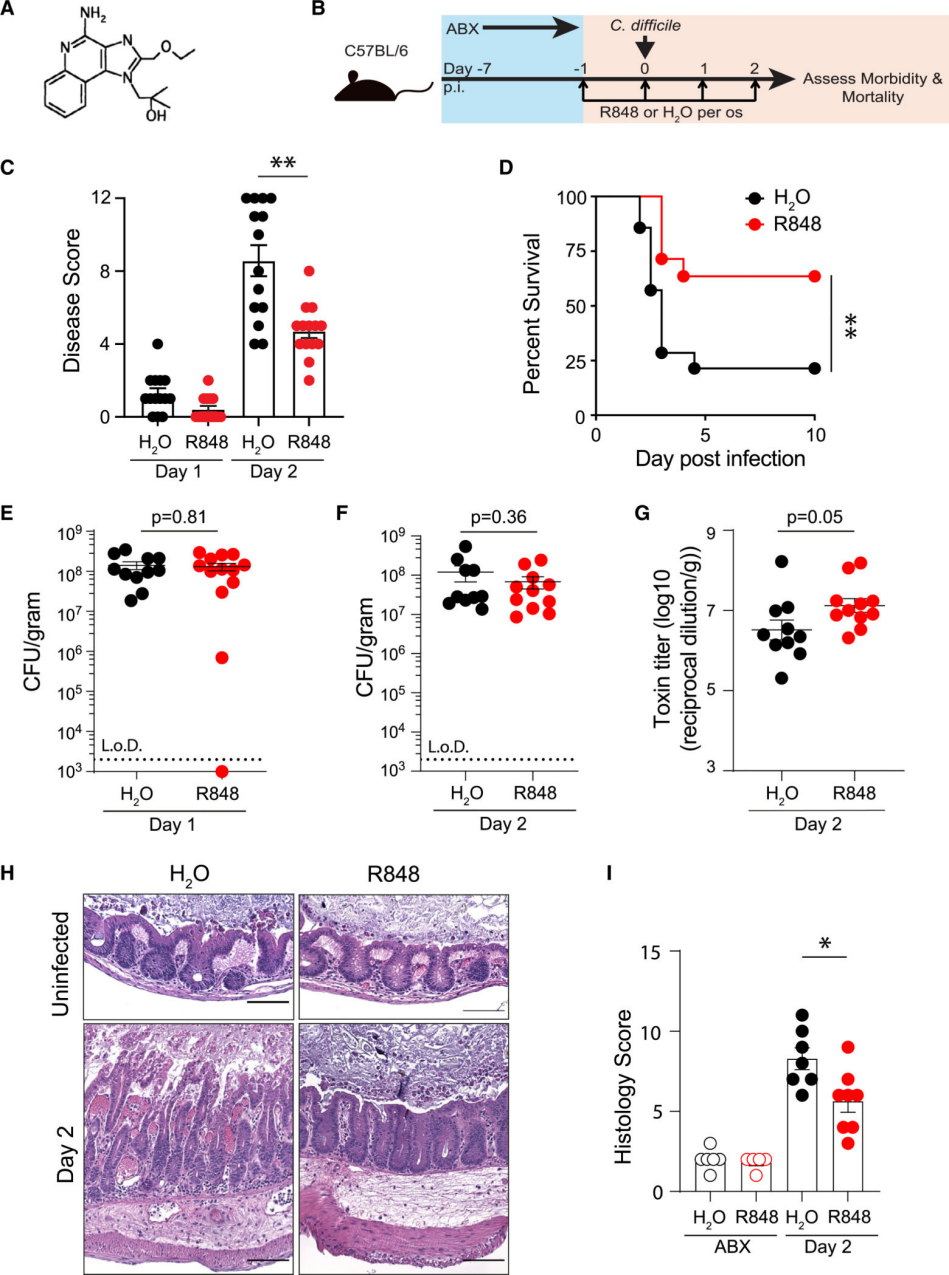
Oral R848 treatment protects mice against severe *C. difficile* infection Antibiotic-treated C57BL/6 mice were orally administered R848 or H_2_O and infected with approximately 200 spores of *C. difficile* (strain VPI 10463). (A) Molecular structure of R848.^33^ (B) Schematic outlining infection experiment. (C and D) (C) Disease severity and (D) survival following infection. Data presented are a combination of three independent experiments (*n* = 14 per treatment group). (E) *C. difficile* burden in stool at day 1. (F and G) (F) *C. difficile* burden and (G) toxin titer in cecal contents at day 2 p.i. Data combined from three independent experiments. (H) Representative images of H&E-stained sections of cecum at day 2 p.i. Scale bar, 100 μm. (I) Histopathological scoring of stained cecal sections based on cell infiltration, epithelial cell death, crypt hyperplasia, and intraluminal exudate (uninfected H_2_O *n* = 6, uninfected R848 *n* = 6, infected H_2_O *n* = 7, infected R848 *n* = 9). Data are presented as mean ± SEM. Statistical significance calculated by (C) Mann-Whitney test, (D) log-rank test, and (E–G and I) unpaired t test. *p < 0.05, **p < 0.01. See also Figures S1–S4.

Epithelial intoxication occurs rapidly following *C. difficile* infection and can elicit an uncontrolled pathogenic inflammatory response that exacerbates intestinal tissue damage.^14,40–42^ Therapeutically augmenting protective immune defenses could limit infection and inflammation-mediated tissue damage. Therefore, we sought to determine whether targeting protective ILC-dependent defenses with R848 (Figure 1A), which was previously reported to activate ILC3s in the ileum,^38^ would enhance host defenses against *C. difficile* infection. Mice were first treated with antibiotics to disrupt the microbiota and induce susceptibility to *C. difficile* (Figure 1B). Following the loss of colonization resistance, mice were orally administered R848 or water (vehicle) daily and orally challenged with *C. difficile* spores (strain VPI 10463) (Figure 1B). This protocol replicates the most common sequence of exposure that leads to *C. difficile* infection in patients. Mice that received vehicle control exhibited severe morbidity at day 2 post infection (p.i.) (Figure 1C) and rapidly succumbed to infection (Figure 1D). In contrast, R848-treated mice exhibited reduced morbidity (Figure 1C) and displayed significant improvement in survival (Figure 1D). Initial studies were conducted with female mice. To confirm that R848-mediated protection was not sex specific, a cohort of male and female C57BL/6 mice were treated with R848 or vehicle and infected with *C. difficile*. R848 treatment reduced the severity of disease at day 2 p.i. (Figure S1A), and there was no difference in protection when stratified by sex (Figure S1B). Interestingly, in contrast to a previous report that R848 treatment reduces pathogenic *Enterococcus* burden in the small intestine,^38^ both treatment groups reached similar levels of *C. difficile* colonization in the feces at day 1 (Figure 1E) and cecal content at day 2 p.i. (Figure 1F). Furthermore, toxin titers were also similar in the cecal content at day 2 p.i. (Figure 1G). Despite equivalent pathogen burden and toxin production, analysis of cecum histology revealed a marked reduction in tissue damage following *C. difficile* infection in mice that received R848 compared to vehicle-treated control mice (Figures 1H and 1I). Furthermore, the primary parameter driving differences in histologic scoring was epithelial cell loss and death (Figure S1C). These results show that R848 treatment boosts the host’s tolerance of *C. difficile* toxin-mediated intestinal epithelial damage.

We next sought to determine the timing of R848 treatment that confers protection (Figure S2A). Mice that received R848 the day before and day of infection (days −1 and 0; ‘‘prophylactic’’) showed no protection from severe disease relative to vehicle-treated control mice (Figure S2B). Mice that received a single dose of R848 at day 1 p.i. (‘‘therapeutic’’) showed reduced morbidity similar to mice that received the full course of R848 (Figure S2A), indicating that R848 can be administered therapeutically following *C. difficile* exposure and still limit the severity of disease.

To begin to uncover the mechanism of R848-mediated protection of the intestinal barrier, the direct effects of R848 on reducing intestinal epithelial cell intoxication was tested (Figure S3). R848 treatment alone did not result in morphological changes to colonic epithelial cell line CaCO_2_ (Figure S3A). Furthermore, CaCO_2_ cells treated with increasing concentrations of R848 and *C. difficile* TcdB showed similar cell rounding relative to cells treated with TcdB alone (Figure S3B), indicating that the protective effect of R848 is not through direct inhibition of *C. difficile* toxin activity.

Susceptibility to *C. difficile* infection is influenced by the composition of the intestinal microbiota at the time of infection,^43^ which may be altered by R848 treatment. To determine whether the protective effects of R848 treatment was supported by changes in microbial composition, we performed 16S rRNA gene sequencing on feces collected on the day of infection (day 0), prior to *C. difficile* spore inoculation. Analysis of weighted UniFrac distances did not delineate samples by treatment groups (Figure S4A), and relative taxonomic abundances appeared similar (Figure S4B), suggesting that the protective effects of R848 are not due to its effects on the intestinal microbiota.

### Protection by R848 is dependent on TLR7 and independent of type I IFN signaling

**Figure 2.**
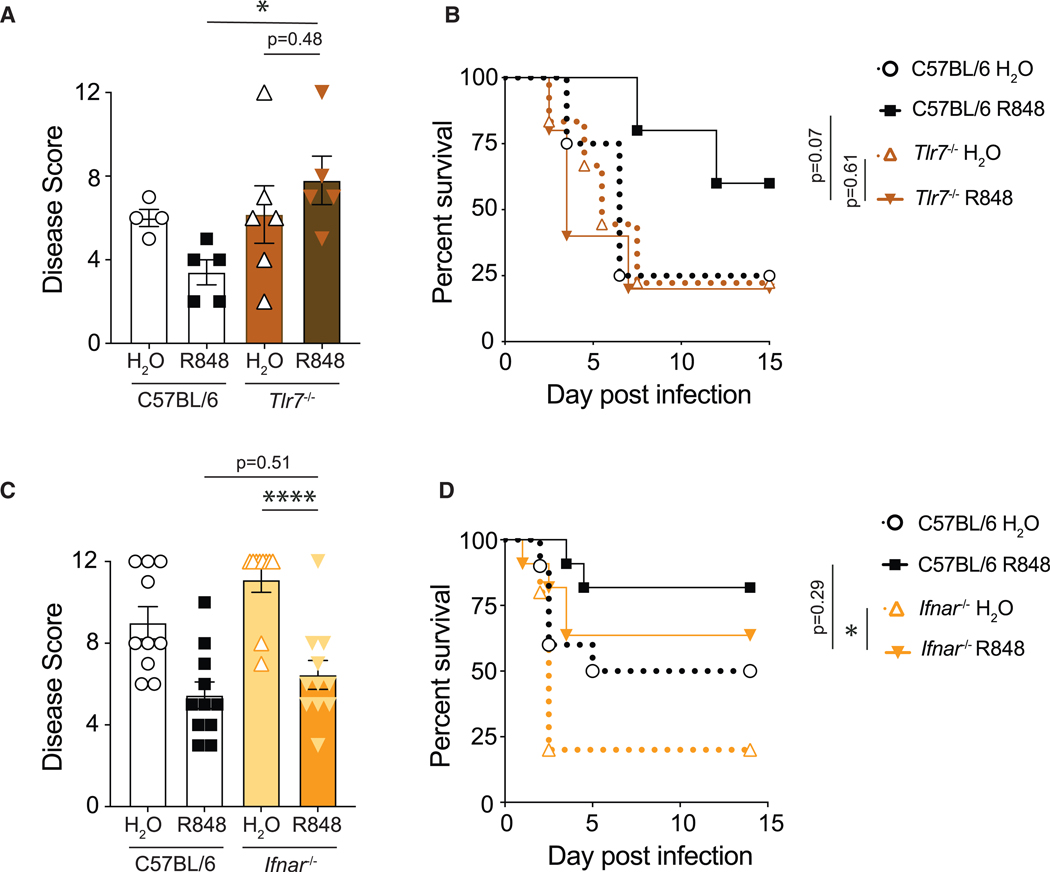
R848-mediated protection is dependent on TLR7 expression but independent of type I IFN signaling Antibiotic-treated mice were administered R848 or H_2_O and infected with *C. difficile*. (A and B) (A) Disease severity at day 2 and (B) survival of R848- or H_2_O-treated C57BL/6 and *Tlr7*^−/−^ mice following infection (*n* = 4–6). (C and D) (C) Disease severity at day 2 and (D) survival of R848- or H_2_O-treated C57BL/6 and *Ifnar*^−/−^ mice following *C. difficile* infection (*n* = 10–11). Data combined from three independent experiments. Data are presented as mean ± SEM. Statistical significance was calculated by (A and C) two-way ANOVA with Šídák’s correction and (B and D) log-rank test. *p < 0.05, **p < 0.01, ***p < 0.001, ****p < 0.0001.

R848 mimics single-stranded viral genomes that canonically induce type I IFN responses downstream of TLR7 and TLR8 signaling.^44,45^ TLR8, however, is not functional in mice.^46^ To confirm that R848 signals through TLR7 to mediate protection, cohoused *Tlr7*^−/−^ and C57BL/6 mice were treated with R848 or vehicle and challenged with *C. difficile*. As expected, while C57BL/6 mice were protected by R848, R848-treated *Tlr7*^−/−^ mice exhibited no difference in disease morbidity (Figure 2A) or mortality (Figure 2B) compared to vehicle-treated *Tlr7*^−/−^ mice, confirming the requirement of this immune receptor in R848-mediated protection. Type I IFNs are predominantly antiviral cytokines and are rapidly induced following R848 treatment of mice.^44,47^ To test whether this canonical pathway contributed to R848-mediated protection against *C. difficile* disease, cohoused C57BL/6 and *Ifnar*^−/−^ mice were treated with R848 or vehicle and challenged with *C. difficile*. Similar to C57BL/6 control mice, R848 treatment protected *Ifnar*^−/−^ mice from severe disease (Figure 2C) and reduced mortality (Figure 2D) following *C. difficile* infection, demonstrating that type I IFN signaling is dispensable for R848-mediated protection.

### R848 treatment activates host innate immune defenses

**Figure 3.**
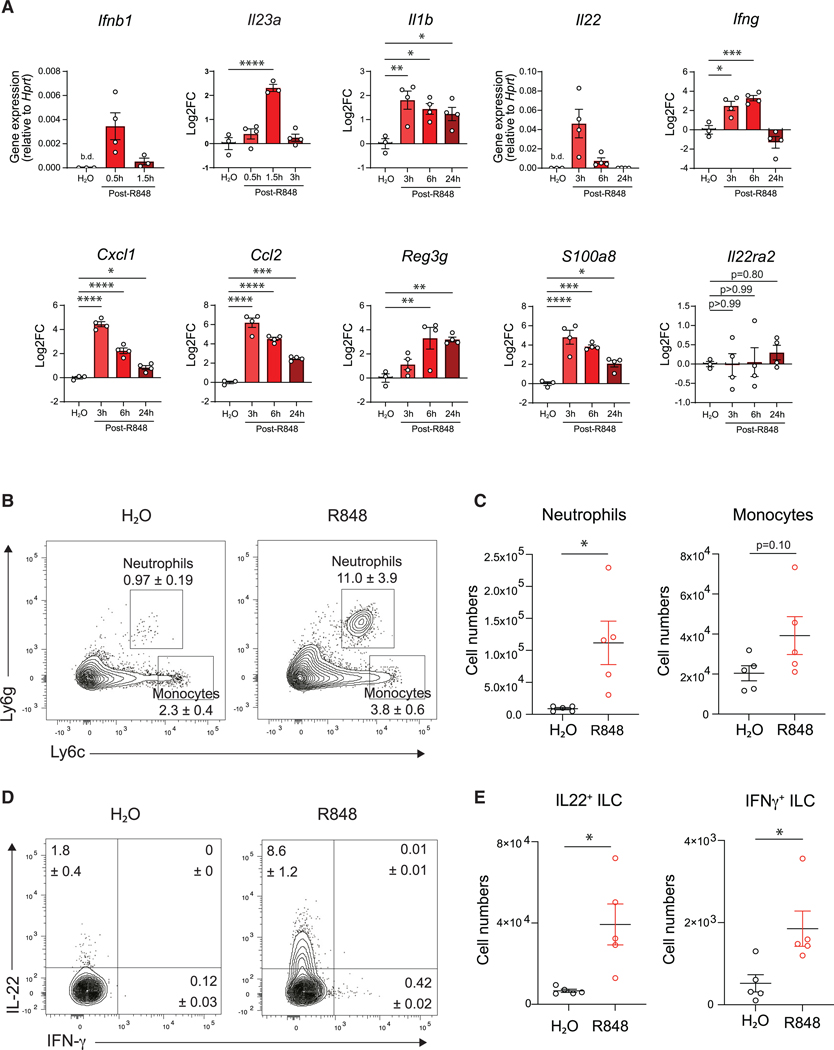
R848 activates immune defenses in the large intestine C57BL/6 mice were treated with R848 or H_2_O and the immune response was assessed. (A) Expression of *Ifnb1* at 0.5 and 1.5 h post-R848; *Il23a* at 0.5, 1.5, and 3 h post-R848; and *Il1b*, *Il22*, *Ifng*, *Cxcl1*, *Ccl2*, *Reg3g*, *S100a8*, and *Il22ra2* at 3, 6, and 24 h post-R848 treatment in whole colon tissue as assessed by RT-qPCR and normalized to *Hprt* expression (*n* = 3–5 per time point). Data expressed as relative gene expression or log2 fold change relative to H_2_O-treated mice and are representative of two experiments. (B and C) (B) Frequency and (C) total number of neutrophils and monocytes in the large intestine lamina propria 3 h post-treatment with R848 or H_2_O (*n* = 5 per group). Cells were gated on live, CD45^+^, CD3^−^, CD5^−^, CD8α^−^, CD19^−^, CD11b^+^ cells. Data representative of two experiments. (D and E) (D) Frequency and (E) total number of ILCs isolated from the large intestine lamina propria at 3 h post-R848 or H_2_O-producing IFN-γ or IL-22 following *ex vivo* incubation in medium with GolgiPlug (BD Biosciences) (*n* = 5 per group). Fluorescence-activated cell sorting (FACS) plots gated on live, CD45^+^, CD3^−^, CD5^−^, CD8α^−^, CD19^−^, Gr-1^−^, CD90^+^, CD127^+^ cells. Data representative of two independent experiments. Data are presented as mean ± SEM. Statistical significance calculated by (A) one-way ANOVA and (C and E) unpaired t test. *p < 0.05, **p < 0.01, ***p < 0.001, ****p < 0.0001. See also Figures S5–S7.

ILCs play a crucial role in the early host response against *C. difficile* infection.^20,21,23,48^ These cells bolster host defenses by producing key cytokines, namely IFN-γ and IL-22, that lead to the recruitment and activation of inflammatory cells and the production of antimicrobial factors. R848 administration was previously shown to stimulate TLR7-expressing CD11c^+^ cells in the small intestine, inducing the expression of IL-23, which leads to IL-22 and downstream induction of antimicrobial Reg3g peptides by the intestinal epithelium.^38^ To determine the immune response elicited by oral administration of R848 in the large intestine prior to infection, we assessed colonic tissue gene expression levels at multiple time points following R848 treatment in uninfected mice. R848 induced robust expression of IFN-β (*Ifnb1*), IL-23 (*Il23a*), IL-1β (*Il1b*), IL-22 (*Il22*), IFN-γ (*Ifng*), and downstream chemokines (*Cxcl1* and *Ccl2*) and antimicrobial peptides (*Reg3g*, *S100a8*) in the large intestine (Figure 3A). The expression of IL-22-binding protein (*Il22ra2*), a negative regulator of IL-22,^49,50^ was not affected by R848 (Figure 3A). Further analysis of the immune cell population revealed significantly greater neutrophil infiltration and a trend toward increased monocytes in the large intestine lamina propria of R848-treated mice at 3 h post-treatment compared to vehicle-treated control mice (Figures 3B, 3C, and S5A), which returned to comparable numbers at 24 h post-R848 treatment (Figures S5B and S5C). Furthermore, staining for intracellular cytokines revealed that R848 treatment induced the expression of IL-22 and IFN-γ specifically in ILCs (Figures 3D, 3E, and S6) but not in CD4^+^ T cells (Figures S7A and S7B) or γδ T cells (Figures S7C and S7D). These experimental data support our model that a dendritic cell-IL-23-ILC-IL-22 signaling axis is occurring throughout the gut-associated lymphoid tissue following R848 stimulation.

### Protection is mediated by IL-22-producing ILCs

**Figure 4.**
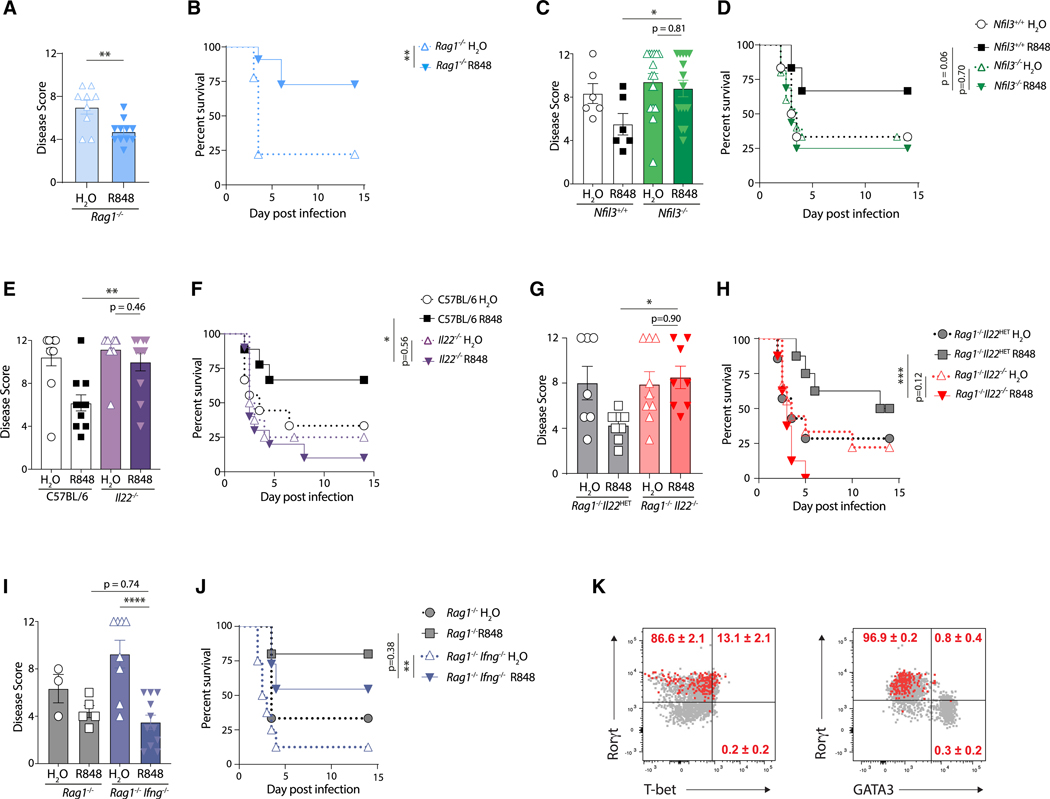
Protection is mediated by IL-22-producing Rorγt-positive innate lymphoid cells (A–J) Antibiotic-treated mice were administered R848 or H_2_O and infected with *C. difficile*. Disease severity at day 2 and survival of R848- or H_2_O-treated (A and B) *Rag1*^−/−^ mice (*n* = 9–11; combined from two experiments), (C and D) *Nfil3*^+/+^ and *Nfil3*^−/−^ mice (*n* = 6–15; combined from four experiments), (E and F) C57BL/6 and *Il22*^−/−^ mice (*n* = 8–11 combined from four experiments), (G and H) *Rag1*^−/−^*IL22*^HET^ and *Rag1*^−/−^*IL22*^−/−^ (*n* = 7–9; combined from two experiments), and (I and J) *Rag1*^−/−^ and *Rag1*^−/−^*Ifng*^−/−^ mice (*n* = 3–11; combined from two experiments). (K) C57BL/6 mice were treated with R848 or H_2_O, and large intestine lamina propria cells were isolated 3 h later and incubated *ex vivo* in medium with Brefeldin A followed by intracellular cytokine and intranuclear transcription factor staining. Rorγt, T-bet, and GATA-3 transcription factor expression in IL-22^+^ ILCs (red) against IL-22^neg^ ILCs (gray) (*n* = 5). Data are presented as mean ± SEM. Statistical significance was calculated by (A) unpaired t test, (C, E, G, and I) two-way ANOVA with Šídák’s correction, and (B, D, F, H, and J) log-rank test. *p < 0.05, **p < 0.01, ***p < 0.001, ****p < 0.0001. See also Figures S8 and S9.

ILC1s and ILC3s are critical in the host defense against *C. difficile* infection, and we observed elevated IL-22 and IFN-γ production from ILCs following R848 (Figures 3D and 3E). We therefore hypothesized that protection by R848 is mediated by ILCs. *C. difficile*-infected *Rag1*^−/−^ mice showed reduced morbidity (Figure 4A) and mortality (Figure 4B) with R848 administration, confirming that T and B lymphocytes are dispensable for R848-mediated protection. To determine whether protection is dependent on ILCs, we utilized *Nfil3*^−/−^ mice, which lack all ILC and natural killer (NK) cell subsets but have functional T and B cells.^23^ R848 treatment protected *Nfil3*^+/+^ control mice but failed to reduce morbidity (Figure 4C) or improve survival (Figure 4D) in *Nfil3*^−/−^ mice following *C. difficile* infection, confirming that R848-mediated protection is dependent on ILCs. Previous studies have shown a protective role for IL-22 during *C. difficile* infection,^26,27,51^ which is induced in the large intestine following R848 administration (Figure 3A). We therefore tested whether IL-22 was necessary for R848-mediated protection. Unlike cohoused C57BL/6 mice that exhibited reduced morbidity and improved survival with R848 treatment compared to vehicle-treated control mice, *Il22*^−/−^ mice treated with R848 showed similar disease severity (Figure 4E) and mortality (Figure 4F) to vehicle-treated *Il22*^−/−^ mice, indicating that the protection afforded by R848 is dependent on IL-22. Analysis of colon histopathology revealed no improvement in *Il22*^−/−^ mice treated with R848 compared to vehicle-treated *Il22*^−/−^ mice (Figures S8A and S8B) and similar epithelial cell loss and death (Figure S8C). To confirm that IL-22 from innate immune cells was critical for R848-mediated protection, *Rag1*^−/−^*Il22*^HET^ and *Rag1*^−/−^*Il22*^−/−^ mice were treated with R848 or vehicle and infected with *C. difficile*. Unlike *Rag1*^−/−^*Il22*^HET^ control mice, R848 treatment failed to reduce morbidity (Figure 4G) or improve survival (Figure 4H) in *Rag1*^−/−^*Il22*^−/−^ mice. IFN-γ producing ILC1s also contribute to early host defense following *C. difficile* infection,^20^ and R848 induced IFN-γ-producing ILCs in the large intestine (Figures 3D and 3E). However, similar to *Rag1*^−/−^ mice, we found R848-treated *Rag1*^−/−^*Ifng*^−/−^ mice exhibited reduced morbidity (Figure 4I) and mortality (Figure 4J) following *C. difficile* infection. To confirm that IL-22^+^ ILCs induced by R848 were ILC3s, ILC-specific transcription factor profiling was conducted and revealed that the IL-22^+^ ILCs in the large intestine lamina propria expressed Rorγt (Figure 4K). Combined, these data indicate that R848 protects by specifically stimulating IL-22-producing Rorγt-expressing ILC3s.

As R848 treatment led to the upregulation of chemokines, rapid recruitment of neutrophils, and ILC activation (Figure 3), we hypothesized that the reduced disease severity was driven by enhanced innate immune activation following *C. difficile* infection. Intriguingly, we observed equivalent induction of inflammatory cytokines, chemokines, and antimicrobial peptide gene expression between R848- and vehicle-treated mice at day 2 p.i. (Figure S9A). *C. difficile* infection leads to pronounced neutrophil and monocyte recruitment to the large intestine lamina propria, helping to clear translocating opportunistic bacteria. Neutrophils in particular have a critical role in the early defense during *C. difficile* toxin-induced colitis.^52,53^ Surprisingly, flow cytometry analysis of neutrophils and monocytes revealed equivalent frequency (Figure S9B) and total numbers (Figure S9C) in the large intestine at day 2 p.i. These results suggest that protection is not driven by effects on infiltrating inflammatory cells. IL-22-producing ILCs were increased in the large intestine lamina propria (Lp) (Figure S9Dand S9E) and mesenteric lymph nodes (mLNs) (Figures S9F and S9G) of R848-treated *C. difficile*-infected mice compared to vehicle-treated mice, further supporting the importance of this cell population in R848-mediated protection.

### IL-22 stimulates intestinal epithelial cell proliferation to restore intestinal barrier integrity following *C. diffizile* infection

The IL-22 receptor is expressed in a broad range of non-hematopoietic tissues, including the gastrointestinal epithelium, liver, thymus, and kidneys. Nagao-Kitamoto et al. showed that the microbiome stimulates IL-22 signaling, which induces epithelial glycosylation, thereby promoting the growth of commensals that outcompete *C. difficile*.^26^ Additionally, Mileto et al. reported that *C. difficile* toxin-mediated epithelial damage was associated with loss of tight-junction proteins and impaired stem cell function.^54^ Therefore, the effects of R848 treatment on epithelial glycosylation and junction gene expression was evaluated in C57BL/6 and *Il22*^−/−^ mice. At 24 h post-treatment, R848 treatment led to decreased expression of host glycosyltransferase genes *Mgat4a*, *Mgat5*, and *St6gal1* (Figure S10A) and the tight-junction protein ZO-1 (encoded by *Tjp1*) (Figure S10B) in an IL-22-dependent manner. These data are consistent with Delbue et al., who found that IL-22 signaling decreases ZO-1 expression in intestinal epithelial cells and suggests that the R848-mediated protection is not driven by increased epithelial glycosylation or junction protein expression.

**Figure 5.**
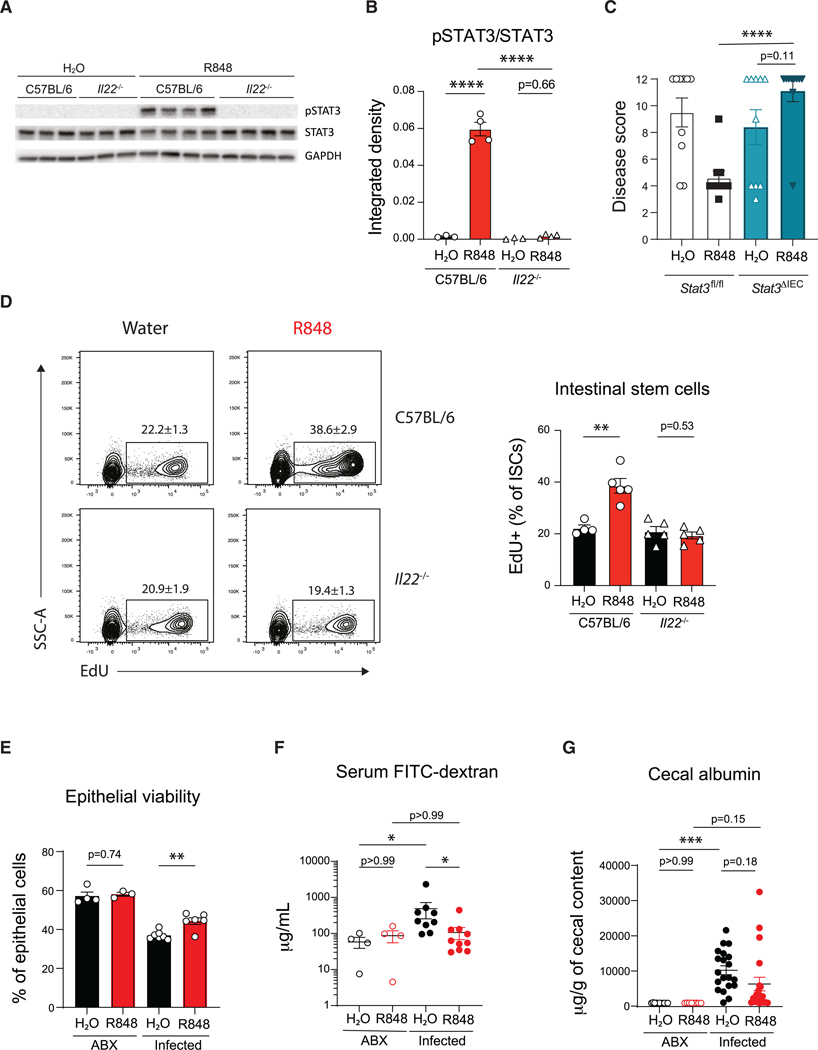
IL-22 signals through STAT3 in intestinal epithelial cells to induce epithelial regeneration and reduce intestinal permeability following *C. difficile* infection Cohoused C57BL/6 and *Il22*^−/−^ mice were treated with R848 or H_2_O and large intestine epithelial fractions were collected 3 h later. (A) STAT3 phosphorylation was determined by immunoblot. (B) STAT3 activation was quantified by dividing the integrated densities of pSTAT3 by STAT3 (*n* = 3–4 per group). (C) Antibiotic-treated *Villin*^Cre^*Stat3*^fl/fl^ mice (*Stat3*^ΔIEC^) and *Stat3*^fl/fl^ (*Stat3*^flox^) controls were treated with R848 or H_2_O and infected with *C. difficile*, and disease severity was assessed at day 2 p.i. Data combined from two experiments (*n* = 10 per group). (D) Frequency of proliferating intestinal stem cells following R848 treatment. Cohoused C57BL/6 and *Il22*^−/−^ mice were treated with R848 or H_2_O and received EdU by intraperitoneal injection 12 h later. Intestinal stem cell proliferation was measured by EdU incorporation 2 h later (*n* = 4–5 per group). FACS plots gated on live, EpCam^+^, CD45^neg^, CD44^hi^, CD24^lo^, c-Kit^neg^ cells to identify intestinal stem cells.^57^ (E–G) Antibiotic-treated C57BL/6 mice were treated with R848 or H_2_O and infected with *C. difficile*, and intestinal barrier integrity was assessed. (E) Epithelial cells (live, CD45^neg^, Epcam^+^) were collected at day 2 p.i., and viability was measured by flow cytometry (*n* = 3–7 per group). (F) Mice received 20 mg of FITC-dextran by oral gavage at day 2 p.i., and intestinal permeability was assessed by measuring serum levels of FITC-dextran 4 h later. Data combined from two experiments (*n* = 4–10 per group). (G) Concentrations of albumin in cecal contents were compared at day 2 p.i. Data combined from four experiments (*n* = 8–20). Data are presented as mean ± SEM. Statistical significance calculated by (B, C, and G) two-way ANOVA with Šídák’s correction, (D and E) multiple t test with Holm-Šidák correction, and (F) Kruskal-Wallis test with Dunn’s correction. *p < 0.05, **p < 0.01, ***p < 0.001, ****p < 0.0001. See also Figures S10–S12.

IL-22 signaling leads to phosphorylation of STAT3 in the colonic epithelium in the context of *C. difficile* infection,^55^ and the protective and regenerative properties of IL-22 are primarily mediated by this transcription factor.^56^ R848 treatment induced STAT3 phosphorylation in large intestine epithelial cells as early as 3 h post-treatment in an IL-22-dependent manner (Figures 5A and 5B). Next, to determine the contribution of intestinal epithelial STAT3 signaling in R848-mediated protection, epithelial-specific STAT3 conditional knockout mice (*Stat3*^ΔIEC^) were generated, treated with R848 or vehicle, and infected with *C. difficile*. While R848 treatment reduced disease severity in control mice (*Stat3*^flox^), R848 failed to ameliorate disease in *Stat3*^ΔIEC^ mice (Figure 5C), indicating that the IL-22 induced by R848 signals on intestinal epithelial cells and activates STAT3-dependent protective mechanisms.

Upon mucosal damage, the intestinal epithelium must undergo repair processes to reestablish barrier function. Since IL-22 signals through STAT3 to support intestinal epithelial regeneration by inducing stem cell proliferation,^56,58^ we treated cohoused C57BL/6 and *Il22*^−/−^ mice with R848 or vehicle and 12 h later assessed stem cell proliferation by 5-ethynyl-2’-deoxyuridine (EdU) incorporation (Figure S11A). R848 treatment significantly increased the proportion of EdU^+^ intestinal stem cells in C57BL/6 mice but not *Il22*^−/−^ mice (Figure 5D). We observed reduced epithelial cell loss in the cecum (Figure S2) and colon (Figure S8C) of R848-treated C57BL/6 mice compared to vehicle-treated control mice by histopathology. Furthermore, a greater proportion of live epithelial cells were recovered from the large intestine of R848-treated mice relative to vehicle-treated control mice at day 2 p.i. as determined by flow cytometry viability staining, supporting our histopathologic observations that R848 treatment limits epithelial damage (Figure 5E). Next, intestinal permeability was measured by quantifying fluorescein isothiocyanate (FITC)-dextran in the serum following oral administration at day 2 p.i. *C. difficile* infection led to an increase in circulating FITC-dextran in vehicle-treated mice that was significantly reduced with R848 treatment (Figure 5F). Serum protein albumin levels were measured in the cecal contents, another measure of intestinal damage. Albumin levels were higher in infected mice compared to uninfected controls, and this was reduced with R848 treatment, although this was not statistically significant (Figure 5G). To investigate the potential of R848 altering vascular permeability, R848- and H_2_O-treated mice were administered Evan’s blue dye retro-orbitally at day 2 p.i.^59^ Huang et al. found that *C. difficile* infection increased colonic vascular permeability.^60^ *C. difficile* infection led to increased Evan’s blue in the kidney relative to uninfected control mice, suggesting increased vascular permeability (Figure S12A). R848 treatment had no effect on vascular permeability in the kidney (Figure S12A) and the cecal tissue (Figure S12B), supporting the hypothesis that the effects of R848 on reducing permeability occur at the epithelial barrier. Together, these findings indicate that R848 limits toxin-induced loss of intestinal barrier function in part by stimulating stem cell proliferation and promoting epithelial survival in an IL-22-STAT3-dependent manner.

### R848 limits systemic disease from disseminated *C. diffizile* toxin

**Figure 6.**
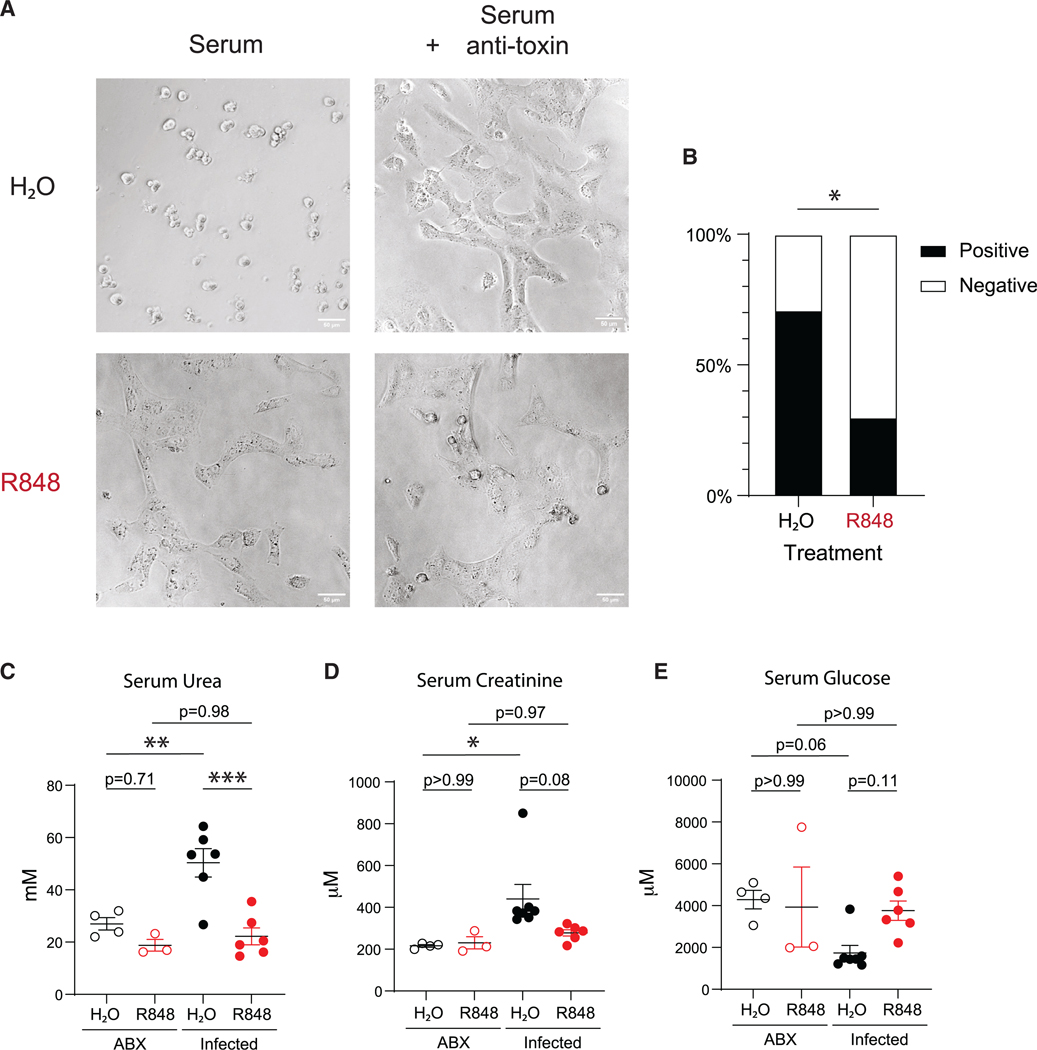
R848 limits toxin dissemination and systemic disease (A) Representative images of Vero cell cultures treated with serum collected from mice at day 2 p.i. (serum) and serum mixed with toxin-neutralizing antisera (serum + anti-toxin). Scale bar, 50 μm. (B) Percentage of mouse sera positive for *C. difficile* toxin. Data combined from four independent experiments (*n* = 17 for H_2_O, *n* = 20 for R848). (C–E) Concentrations of (C) urea, (D) creatinine, and (E) glucose in serum collected from mice at day 2 p.i. (*n* = 3–6). Data are presented as mean ± SEM. Statistical significance was calculated by (B) Fisher’s exact test and (C–E) two-way ANOVA with Šídák’s multiple comparison correction. *p < 0.05, **p < 0.01, ***p < 0.001.

The systemic dissemination of *C. difficile* toxins TcdA and TcdB is associated with severe disease.^10^ Thus, we assessed the effects of R848 treatment on circulating toxin to determine how R848 treatment improved host survival following *C. difficile* infection. Serum collected from *C. difficile*-infected mice at day 2 p.i. was assessed for the presence of toxin by cell rounding of cultured Vero cells. We found *C. difficile* toxin was detected at a lower frequency in mice that received R848 (30%) compared to vehicle (71%) (Figures 6A and 6B). Toxemia during *C. difficile* infection leads to systemic complications and damage to extraintestinal organs.^13,61^ We therefore compared circulating levels of markers for systemic disease. We assessed serum levels of urea and creatinine, two markers of kidney function. We found the serum levels of urea that were increased with *C. difficile* infection were significantly reduced in mice that received R848 treatment at day 2 p.i. (Figure 6C). Additionally, serum levels of creatinine that were increased with infection were also reduced in R848-treated mice, although this was not statistically significant (Figure 6D). Bacterial infections can result in hypoglycemia from hepatic dysfunction or sepsis.^62–64^ We found that *C. difficile* infection led to a decrease in serum levels of glucose, and this trended higher with R848 treatment (Figure 6E). Together, our findings indicate that R848 treatment promotes intestinal barrier integrity, limiting the dissemination of *C. difficile* toxin, thereby preventing systemic disease.

## DISCUSSION

Immune defense comprises not only resistance mechanisms that reduce pathogen burden or alter virulence factor expression but also tolerance mechanisms that enable the host to adapt to a pathogen’s presence without inducing collateral immunopathology.^15–18^ *C. difficile* is adapted to the luminal environment of the large intestine and colonizes at high burdens even in the presence of host immune pressures, but the pathogen does not disseminate into host tissue. Therefore, effective immune defenses against *C. difficile* infection must indirectly counter the pathogen by promoting intestinal tissue tolerance of toxin-mediated damage in addition to resistance mechanisms that clear opportunistic intestinal bacteria that can translocate across the damaged epithelium.^21,25,52^ This study demonstrates that therapeutically stimulating the IL-22 axis allows the host to tolerate mucosal injury following *C. difficile* infection. These host tolerance measures can provide time for the disrupted microbiome to recover and directly eliminate *C. difficile* from the intestinal tract.

*C. difficile* infection leads to the loss of intestinal epithelial cells and impaired barrier function. The ensuing dissemination of *C. difficile* toxins has been implicated as a contributor to systemic complications and severe disease.^10–12^ Thus, identifying ways to promote mucosal repair or enhance tissue tolerance to toxin-mediated injury to the epithelium is a promising target to mitigate morbidity. Here, we report that administering the TLR7 agonist resiquimod (R848) transiently activates innate immune defenses and increases host tolerance to *C. difficile* infection without collateral tissue damage from prolonged intestinal inflammation. R848 treatment reduced mucosal damage from infection and prevented the dissemination of *C. difficile* toxin, thereby limiting their effects on systemic organ function. We show that this protection was mediated by ILCs producing IL-22, which signals on intestinal epithelial cells to activate STAT3-dependent regenerative pathways. A previous study demonstrated that TLR7-expressing CD11c^+^ dendritic cells responded to R848 and produced IL-23, which stimulated IL-22 production from ILCs to reduce colonization by vancomycin-resistant enterococcus.^38^ Our work suggests that R848-mediated IL-22 stimulation is protective against a wide range of enteric pathogens, although, in this work, pathogen burden was not affected and instead R848 stimulation promoted host tissue tolerance of toxin-mediated damage.

Several mechanisms for the protective role of IL-22 during *C. difficile* infection have been described. IL-22 induces complement protein expression in the liver, and the lack of IL-22 signaling leads to greater dissemination of translocating commensal bacteria following infection.^25^ However, in contrast to our study, this report observed no significant differences in intestinal damage, suggesting that therapeutically enhancing IL-22 expression via R848 treatment protects through a different mechanism. Additionally, modulation of host epithelial glycosylation by IL-22 promotes the growth of commensal bacteria that outcompete *C. difficile* for succinate, a metabolite that fuels *C. difficile* growth in the intestine.^26,65^ Furthermore, a recent study reported that *Il22ra2*^−/−^ mice that lack IL-22-binding protein, the soluble decoy receptor that sequesters IL-22, select for intestinal microbes that protect against *C. difficile* infection.^50^ While we observed altered expression of glycosyltransferase genes with R848, we saw no significant differences in microbiota composition at the time of infection, nor did we observe differences in the levels of *C. difficile* colonization between treatment groups, suggesting that protection by R848 treatment is not due to modulation of epithelial glycosylation or altered competition between the microbiota and the pathogen.

The intestinal epithelium undergoes constant renewal with epithelial turnover occurring every 5–7 days in the colon.^66^ Upon injury, epithelial regeneration can be induced to repair the damaged intestinal barrier.^67^ IL-22 stimulates epithelial cell proliferation through STAT3 signaling and facilitates tissue repair following intestinal damage.^56,58,68^ IL-22 activation also promotes cell survival by regulating genes that protect against apoptosis^56^ and stimulates the migration of intestinal epithelial cells with the potential to support mucosal healing.^69,70^ Through EdU incorporation analysis and viability staining, we found R848 stimulates the proliferation of intestinal stem cells and promotes the survival of epithelial cells following infection. Thus, R848-induced IL-22 facilitates repair of the epithelium following toxin-induced damage. IL-22 also regulates the expression of epithelial junction genes; however, we found R848 led to altered expressions consistent with increased permeability and barrier dysfunction. Finally, IL-22 also plays an important role in maintaining tolerance to the intestinal microbiota by regulating the production of membrane mucus and antimicrobial peptides.^71–73^ While we noted no attenuation of *C. difficile* colonization with R848 treatment or differences in microbiota composition between treatment groups, differences in commensal bacteria metabolism may support epithelial responses that contribute to reduced disease severity in R848-treated mice. Thus, multiple mechanisms downstream of IL-22 signaling likely work in concert to protect against enteric infections.^74^

### Limitations of the study

While R848 treatment did not lead to observable pathologic signs, the broad activation of the innate immune system could have potentially adverse effects. For example, chronic inflammation in patients with inflammatory bowel disease (IBD) is a known risk factor for *C. difficile* infection and is associated with higher mortality than individuals without IBD.^75^ The IL-23 pathway, which is induced by R848 and signals upstream of IL-22, plays a key role in the pathogenesis of IBD, and antibodies that block IL-23 are used to treat IBD.^76^ Thus, a more targeted approach that specifically stimulates the IL-22 signaling pathway without activation of other pathways downstream of TLR7 may be suitable for mitigating the severity of *C. difficile* infection. Recently developed delivery systems such as IL-22 delivery through recombinant fusion protein,^77^ mRNA lipid nanoparticle,^78^ or engineered bacterium^79,80^ could provide a promising novel therapeutic approach.

## RESOURCE AVAILABILITY

### Lead contact

Further information and requests for resources and reagents should be directed to the lead contact, Michael C. Abt (Michael.abt@pennmedicine.upenn.edu).

### Materials availability

All materials generated in this study will be made available upon reasonable request.

### Data and code availability

- The 16S rRNA gene sequencing data have been deposited at NCBI SRA database as BioprojectID: PRJNA1133716 and are publicly available as of the date of publication. The flow cytometry data have been deposited at Mendeley Data and are publicly available as of the date of publication at Mendeley Data: https://doi.org/10.17632/npwvj89b6m.1.
- This paper does not report original code.
- Any additional information required to reanalyze the data reported in this paper is available from the lead contact upon request.

## STAR★METHODS

### EXPERIMENTAL MODEL AND STUDY PARTICIPANT DETAILS

#### Mice

All knockout strains are on C57BL/6 background. *Rag1*^−/−^*Il22*^−/−^ mice and *Rag1*^−/−^*Ifng*^−/−^ mice were generated by crossing *Rag1*^−/−^ mice to *Il22*^−/−^ and *Ifng*^−/−^ mice, respectively. *Villin*^Cre^*Stat3*^*fl/fl*^ was generated by crossing *Villin*^Cre^ mice to *Stat3*^fl/fl^ mice. All mice were bred and maintained in autoclaved cages on LabDiet 5010 autoclaved rodent diet and autoclaved water under specific pathogen-free conditions at the University of Pennsylvania. Where possible, adult mice (at least 8 weeks old) of both sexes were used in experiments. In experiments where knockout strains were cohoused with C57BL/6 mice, male mice were combined before 5 weeks of age to avoid fighting. All procedures were approved by the Institutional Animal Care and Use Committee of the University of Pennsylvania.

#### Cell lines

All cell lines were purchased from ATCC and were verified mycoplasma negative. Vero cells (ATCC CCL-81) were cultured in Dulbecco’s Modified Eagle Medium (Corning) supplemented with 10% FBS, 100 U/mL penicillin (Gibco), 100 μg/mL streptomycin (Gibco), 50 μg/mL gentamicin (Gibco), 10 mM HEPES (Cytiva), 0.5 mM β-mercaptoethanol (Gibco), 1 mM sodium pyruvate (Sigma-Aldrich), and 20 μg/mL L-glutamine (Corning) and incubated at 37°C with 5% CO_2_. Caco-2 cells (ATCC HTB-37) were cultured in Dulbecco’s Modified Eagle Medium (Corning) supplemented with 20% FBS, 100 U/mL penicillin (Gibco), and 100 μg/mL streptomycin (Gibco) and incubated at 37°C with 5% CO_2_.

#### Bacterial strains

*C. difficile* strain VPI 10463 was purchased from ATCC (#43255). PCR Ribotyping of the 16S gene region was performed to confirm VPI 10463 strain. To prepare spores for infection, single colonies of *C. difficile* were grown anaerobically in brain heart infusion broth (BD Biosciences) supplemented with 5 g/L yeast extract (BD Biosciences), 1 g/L L-cysteine (Sigma-Aldrich), 0.25 g/L D-cycloserine (Sigma-Aldrich), and 8 mg/L cefoxitin (Sigma-Aldrich) (BHIS) for at least 28 days to promote sporulation. To isolate spores, cells were pelleted and washed with sterile water. HistoDenz (Sigma-Aldrich) gradient was used to separate spores from vegetative cells. After HistoDenz separation, spore fraction was incubated at 65°C for 20 min to kill residual vegetative cells. Spores were then washed and resuspend in sterile water. Spore purity was verified by plating on BHIS agar.

### METHOD DETAILS

#### *C. diffizile* infection and R848 treatment

Mice of different strains were cohoused for at least three weeks prior to the start of the experiment and remained cohoused for the duration of the infection to normalize the microbiota. To induce susceptibility to *C. difficile* infection, mice received antibiotic water consisting of 0.25 g/L metronidazole (Sigma-Aldrich), 0.33 g/L neomycin (Sigma-Aldrich), and 0.33 g/L vancomycin (Novaplus) for 4 days, then 200 mg of clindamycin (Sigma-Aldrich) by intraperitoneal injection two days following cessation of antibiotic water. The following day, mice were infected with approximately 200 spores of *C. difficile* (Strain VPI 10463, ATCC 43255) by oral gavage. Mice were monitored daily following infection and disease severity was scored according to four parameters: weight loss (>95% of initial weight, 0; 90–95%, 1; 80–90%, 2; <80%, 3), body temperature (>32°C, 0; 31°C–32°C, 1; 30°C–31°C, 2; <30°C, 3), stool consistency (formed pellet, 0; loose pellet, 1; liquid, 2; wet tail, 3), and body condition (1 for each symptom; ruffled fur, hunched, lethargic, and ocular discharge). Mice with severe disease defined as weight loss greater than 30% or body temperature below 29.5°C were humanely euthanized by CO_2_ asphyxiation.

R848 (Invivogen) was reconstituted to 1 mg/mL in sterile endotoxin-free water and stored at −20°C. For treatment of mice, 50 μg was administered daily in 200 μL by oral gavage starting the day before *C. difficile* infection and continuing to day 2 post-infection (p.i.).

#### Quantification of pathogen burden

To quantify bacterial load, fecal pellets or intestinal contents were resuspended in 1 mL of deoxygenated PBS and serial 10-fold dilution was plated on brain heart infusion agar (BD Biosciences) supplemented with 5 g/L yeast extract (BD Biosciences), 1 g/L L-cysteine (Sigma-Aldrich), 0.25 g/L D-cycloserine (Sigma-Aldrich), 8 mg/L cefoxitin (Sigma-Aldrich), and 1 g/L taurocholic acid (MP Biomedicals) and incubated anaerobically at 37°C.

#### Quantification of *C. diffizile* toxin titers

Suspensions from pathogen burden quantification was centrifuged and supernatants were subsequently used to quantify toxin in intestinal contents. To measure toxin titers, Vero cells were seeded in 96-well TC-treated plates at 2 × 10^4^ cells. The following day, 10-fold serially diluted samples were added to cells and cell morphology was assessed 24 h later. The highest dilution where cell-rounding was still observed was used to calculate toxin titers. Cytotoxicity by *C. difficile* toxin was confirmed by neutralization with anti-toxin antisera (Techlab). For detection of circulating toxin, blood was collected by cardiac puncture and serum was collected using Microtainer Blood Collection Tubes (Becton Dickinson) according to manufacturer protocol. Collected serum was subsequently used in cytotoxicity assay without serial dilution as described above. Images were acquired on a Ti-2 inverted microscope (Nikon) using 10×/0.3 objective and DS-Fi3 camera (Nikon). Images were acquired using NIS-Elements AR software (Nikon, v. 5.20.01) and processed with ImageJ software (NIH).

#### Intoxication of intestinal epithelial cells

To determine the direct effects of R848 on intestinal epithelial intoxication, Caco-2 cells were seeded in 96-well plates at 3 × 10^4^ cells. The following day, cells were treated with serial 10-fold dilutions of R848 (0.005–50 μg/mL) or media and subsequently intoxicated with 20 or 100 p.m. of purified TcdB. The next day, cell-rounding was assessed as an indicator of intoxication. Images were acquired on a Ti-2 inverted microscope (Nikon) using 10×/0.3 objective and DS-Fi3 camera (Nikon). Images were acquired using NIS-Elements AR software (Nikon, v. 5.20.01) and processed with ImageJ software (NIH).

#### Isolation of immune cells and flow cytometry

The large intestine (cecum and colon) was harvested, opened longitudinally, and contents removed with PBS with 1% fetal bovine serum (FBS; GeminiBio). To remove intestinal epithelial cells, tissues were incubated in strip buffer (PBS, 5 mM EDTA, 1 mM dithiothreitol, 4% FBS, and 10 μg/mL penicillin-streptomycin) with agitation at 37°C for 10 min. Incubation was repeated with fresh strip buffer for 20 min to remove intraepithelial lymphocytes. To obtain immune cells from the lamina propria, the tissue was next digested with 1.25 mg/mL collagenase IV (Worthington Biochemical) and 20 μg/mL DNAse I (Sigma-Aldrich) in Dulbecco’s Modified Eagle Medium (Corning) supplemented with 10% FBS (GeminiBio), 200 μg/mL L-glutamine (Corning), 1 mM sodium pyruvate (Sigma-Aldrich), 20 mM HEPES (Cytiva), 55 μM 2-mercaptoethanol (Gibco), 50 μg/mL gentamicin sulfate (GeminiBio), and 100 U/mL penicillin (Gibco), 100 μg/mL streptomycin (Gibco) for 30 min. Suspensions were passed through a 100 μm strainer followed by a 40 μm strainer to obtain single cells. Cells were quantified on a Cellometer Auto 2000 (Nexcelom). 1–2 × 10^6^ cells were plated in 96-well round-bottom plates and stained with LIVE/DEAD Fixable Aqua Dead Cell Stain (Invitrogen) for 20 min. For intracellular cytokine staining, cells were plated in 96-well plates and incubated with GolgiPlug (BD Biosciences) for 4 h at 37°C prior to dead cell staining. Positive controls were incubated in the presence of PMA/Ionomycin (Sigma-Aldrich) or recombinant murine IL-23 (Biolegend). Cells were subsequently blocked with 0.1 mg/mL anti-mouse CD16/32 (clone 2.4G2; BD Biosciences) and 0.2 mg/mL rat IgG (Sigma-Aldrich) and stained with fluorescently conjugated antibodies. For intracellular cytokine and transcription factor staining, cells were fixed and permeabilized using Foxp3/Transcription Factor staining set (eBiosciences) followed by antibody staining. Data was collected on an LSR-II or FACSSymphony A3 flow cytometer (BD Biosciences) and analyzed using FlowJo software (v. 10.10.0).

#### Intestinal epithelial proliferation assays

To measure intestinal stem cell proliferation, mice were intraperitoneally injected with 1 mg of 5-Ethynyl-2′-deoxyuridine (EdU), and large intestine epithelial cells were isolated 2 h later as described above. Following viability and surface marker staining, cells underwent click chemistry to label EdU with Alexa Fluor 488 for flow cytometry detection following manufacturer protocol (Invitrogen).

#### Tissue RNA isolation, cDNA preparation, and qRT-PCR

1–2 cm of the proximal colon was harvested and stored in RNAlater at −80°C until RNA extraction was performed. RNA was isolated from proximal colon using mechanical disruption and RNeasy mini kit (Qiagen). RNA samples were reverse transcribed using the QuantiTect Reverse Transcription kit (Qiagen). Quantitative reverse transcription-PCR (qRT-PCR) was performed using either TaqMan primers and probes with the DyNamo ColorFlash Probe qPCR Kit (Thermo Scientific) or QuantiTect primers with QuantiNova SYBR Green PCR Kit (Qiagen) on a QuantiStudio 6 Flex Real-Time PCR system (Applied Biosystems). Gene expression was normalized to the reference gene *Hprt,* and data is expressed as fold difference over water-treated controls.

#### Bacterial DNA isolation and 16S rRNA sequencing

DNA was extracted from fecal pellets using the DNeasy PowerSoil Pro kit (Qiagen). Sequencing library was prepared by amplifying the V4-V5 region of the bacterial 16S rRNA gene using the dual-indexing approach as described previously^81^ and sequenced on the Illumina MiSeq platform. Sequencing data was demultiplexed using the fqgrep tool, imported into QIIME2 (v. 2020.2),^82^ and denoised using the DADA2 plugin^83^ in R (v. 3.6.3).^84^ Taxonomic classification was done in QIIME2 using the q2-feature-classifier^85^ classifysklearn naive Bayes classifier with a newly generated classifier against Greengenes 13_8 99% operational taxonomic unit (OTU) sequences.^86^ Phylogenetic trees were created using mafft^87^ and the q2-phylogeny plugin.^88^ Data were then imported into R for further analyses using phyloseq (v. 1.30.0)^89^ and plotted with ggplot2 (v. 3.3.0).^90^ Weighted UniFrac^91^ dissimilarity was calculated to generate plots of principal coordinate analysis.

#### Isolation of epithelial cells and immunoblot analysis

The large intestine (cecum and colon) was harvested, opened longitudinally, and contents removed with PBS with 1% fetal bovine serum (FBS; GeminiBio). Epithelial cells were collected by incubating tissues in strip buffer (PBS, 5 mM EDTA, 1 mM dithiothreitol, 4% FBS, and 10 μg/mL penicillin-streptomycin) with agitation at 37°C for 10 min and filtering through 100 μm cell strainers. Cells were pelleted and stored at −80°C.

For immunoblotting, cell pellets were first resuspended in RIPA buffer (150 mM NaCl, 1% Triton X-100, 0.5% sodium deoxycholate, 0.1% SDS, and 50 mM Tris) with protease inhibitor (Roche) to extract protein. Protein concentrations were measured using Pierce BCA assay. Samples were normalized to 1 mg/mL and 10 μL was run on 4–15% pre-cast gels (BioRad) and transferred to PVDF membranes (BioRad). Membranes were blocked with blocking buffer (Tris buffer with 0.1% Tween 20 and 5% nonfat dry milk) and incubated with primary antibodies overnight in Tris buffer with 0.1% Tween 20 and 5% BSA. The following day, membranes were incubated with secondary antibody in blocking buffer and developed with Pierce ECL (Thermo Scientific). Membranes were imaged on an Amersham Imager 600 (GE Life Sciences). Membranes were stripped using stripping buffer (200 mM glycine, 3.5 mM SDS, 1% Tween 20, pH 2.2) and re-probed with multiple primary antibodies.

#### Intestinal permeability measurement

At day 2 p.i., food and bedding were removed for 3 h prior to administering 20 mg of 4 kDa fluorescein isothiocyanate (FITC) dextran (Sigma-Aldrich) by oral gavage. Blood was collected by cardiac puncture 4 h later, and fluorescence was measured in the serum using an EnVision Multimode Plate Reader (PerkinElmer). The concentration of FITC-dextran in serum was determined using a standard curve after correcting for background fluorescence of serum.

The concentration of albumin was determined in intestinal contents used to enumerate pathogen burden with Mouse Albumin ELISA kit (Fortis Life Sciences) following manufacturer instructions. Samples were run in technical duplicate and plates were analyzed on a SpectraMax 190 microplate reader (Molecular Devices).

#### Vascular permeability assessment

At day 2 p.i., mice were intravenously injected with 100 μL of 0.5% solution of Evans blue dye in PBS 30 min before the cecum and kidney were harvested. Tissues were then weighed, and Evans blue dye was extracted by incubating in 500 μL of formamide for 24 h at 55°C.^59^ The extracted dye solution was centrifuged to remove residual tissue fragments before measuring absorbance at 610 nm on a SpectraMax 190 microplate reader. The concentration of Evans dye per mg tissue was calculated according to a standard curve.

#### Serum assays

Concentrations of urea in serum samples were measured using a urea assay kit (Abcam) according to manufacturer instructions. To measure the concentrations of creatinine and glucose, serum samples were first deproteinized with perchloric acid (1M final concentration) and neutralized with potassium hydroxide. Creatinine and glucose concentrations were determined in deproteinized samples using commercial assay kits (Abcam). Samples were run in technical duplicate.

#### Histopathologic scoring

The ceca and proximal colon were harvested and fixed in 10% formalin. Sections were stained with hematoxylin and eosin and scored blind by the Comparative Pathology Core in the University of Pennsylvania School of Veterinary Medicine or by Dr. Emma Furth in the Department of Pathology of the University of Pennsylvania Medical Center. Slides were scored on a scale of 0–4 in each of the following categories: inflammatory cell infiltration, epithelial cell death, crypt hyperplasia, and intraluminal exudate.

### QUANTIFICATION AND STATISTICAL ANALYSIS

Data is presented as mean ± SEM. Statistical significance was calculated as indicated in the figure legends. *p < 0.05, **p < 0.01, ***p < 0.001, ****p < 0.0001. Statistical analyses were performed using Prism software (GraphPad; v. 10.1.0).

## Supplementary Material

1

## Figures and Tables

**Table:** KEY RESOURCES TABLE

REAGENT or RESOURCE	SOURCE	IDENTIFIER
Antibodies		

Marker	Company	Catalog #
Anti-mouse CD16/32, clone 2.4G2	BD Biosciences	Cat# 553142; RRID: AB_394656
Rat IgG	Sigma Aldrich	Cat# I4131; RRID: AB_1163627
Anti-mouse CD45, BV605, clone 30-F11	Biolegend	Cat# 103155; RRID: AB_2650656
Anti-mouse CD3, PerCP Cy5.5, clone 1452C11	eBioscience	Cat# 45-0031-82; RRID: AB_1107000
Anti-mouse CD5, PerCP Cy5.5, clone 53-7.3	eBioscience	Cat# 45-0051-82; RRID: AB_914334
Anti-mouse CD19, BV785, clone 6D5	Biolegend	Cat# 115543; RRID: AB_11218994
Anti-mouse CD11b, PE-eFluor610, clone M1/70	eBioscience	Cat# 61-0112-82; RRID: AB_2574528
Anti-mouse CD11c, PE Cy7, clone: N418	eBioscience	Cat# 25-0114-82; RRID: AB_469590
Anti-mouse Ly6c, eF780, clone HK1.4	eBioscience	Cat# 47-5932-82; RRID: AB_2573992
Anti-mouse Ly6g, AF700, clone 1A8	Biolegend	Cat# 127622; RRID: AB_10643269
Anti-mouse CD170 (Siglec-F), APC, clone S17007L	Biolegend	Cat# 155508; RRID: AB_2750236
Anti-mouse CD45, AF700, clone 30-F11	Biolegend	Cat# 103128; RRID: AB_493715
Anti-mouse CD4, BV605, clone RM4-5	Biolegend	Cat# 100548; RRID: AB_11125962
Anti-mouse CD8a, PerCP Cy5.5, clone 53-6.7	eBioscience	Cat# 45-0081-82; RRID: AB_1107004
Anti-mouse CD19, BV650, clone 6D5	Biolegend	Cat# 115541; RRID: AB_11204087
Anti-mouse gdTCR, APC Fire 750, clone GL3	Biolegend	Cat# 118136; RRID: AB_2650828
Anti-mouse NK1.1, BV785, clone PK136	Biolegend	Cat# 108749; RRID: AB_2564304
Anti-mouse Gr-1, PE-Dazzle, clone RB6-8C5	Biolegend	Cat# 108452; RRID: AB_2564249
Anti-mouse CD90.2, eF450, clone 53-2.1	eBioscience	Cat# 48-0902-82; RRID: AB_1272200
Anti-mouse CD127, PE Cy7, clone A7R34	eBioscience	Cat# 25-1271-82; RRID: AB_469649
Anti-mouse IFN-g, APC, clone XMG1.2	eBioscience	Cat# 17-7311-82; RRID: AB_469504
Anti-mouse IL-22, PE, clone 1H8PWSR	eBioscience	Cat# 12-7221-82; RRID: AB_10597428
Anti-mouse Rorgt, APC, clone B2D	eBioscience	Cat# 17-6981-82; RRID: AB_2573254
Anti-mouse T-bet, BV421, clone 4B10	Biolegend	Cat# 644815; RRID: AB_2686976
Anti-mouse GATA-3, PerCP eF710, clone TWAJ	eBioscience	Cat# 46-9966-42; RRID: AB_10804487
Anti-mouse CD45, AF700, clone 30-F11	Biolegend	Cat# 103128; RRID: AB_493714
Anti-mouse Epcam, PerCP Cy 5.5, clone G8.8	Biolegend	Cat# 118220; RRID: AB_2246499
Anti-mouse CD44, APC, clone IM7	Biolegend	Cat# 103012; RRID: AB_312963
Anti-mouse CD24, BV605, clone M1/69	Biolegend	Cat# 101827; RRID: AB_2563464
Anti-mouse CD117 (c-kit), BV785, clone 2B8	Biolegend	Cat# 105841; RRID: AB_2629799
Rat IgG1, APC, clone EBRG1	eBioscience	Cat# 17-4301-82; RRID: AB_470178
Rat IgG1, PE, clone EBRG1	eBioscience	Cat# 12-4301-82; RRID: AB_470047
Anti-mouse pSTAT3 (Tyr705), Rabbit	Cell Signaling	Cat# 9145; RRID: AB_2491009
Anti-mouse STAT3, Rabbit	Cell Signaling	Cat# 4904; RRID: AB_331269
Anti-mouse GAPDH, Rabbit	Cell Signaling	Cat# 2118; RRID: AB_561053
Anti-rabbit IgG, HRP-linked	Cell Signaling	Cat# 7074; AB_2099233

Bacterial and virus strains		

*Clostridioides difficile*, strain VPI 10463	ATCC	43255

Chemicals, peptides, and recombinant proteins		

Metronidazole	Sigma-Aldrich	M1547
Neomycin	Sigma-Aldrich	N1876
Vancomycin	Fresenius Kabi	314061
Clindamycin	Sigma-Aldrich	PHR1159
Resiquimod (R848)	Invivogen	tlrl-r848
Brain heart infusion agar	BD Biosciences	237500
Yeast extract	BD Biosciences	212750
L-cysteine	Sigma-Aldrich	C7352
D-cycloserine	Sigma-Aldrich	C6880
Cefoxitin	Sigma-Aldrich	C4786
Taurocholic acid	MP Biomedicals	0210293025
HistoDenz	Sigma-Aldrich	D2158
Delbecco’s Modified Eagle Medium	Corning	10-017-CV
Fetal Bovine Serum	GeminiBio	100-500
Penicillin-Streptomycin	Gibco	15-140-122
Gentamicin	GeminiBio	400-108
HEPES	Cytiva	SH30237.01
β-mercaptoethanol	Gibco	21985023
Sodium pyruvate solution	Sigma-Aldrich	S8636
L-glutamine	Corning	25-005-CI
Anti-toxin antisera	Techlab	T5000
EDTA	Invitrogen	15575-038
Dithiothreitol	Fisher Scientific	BP172
Collagenase IV	Worthington Biochemical	LS004189
DNAse I	Sigma-Aldrich	10104159001
LIVE/DEAD Fixable Aqua Dead Cell Stain	Invitrogen	L34966
GolgiPlug	BD Biosciences	554714
Phorbol 12-myristate 13-acetate	Sigma-Aldrich	P8139
Ionomycin	Sigma-Aldrich	I0634
Recombinant murine IL-23	Biolegend	589004
Foxp3/Transcription Factor staining buffer set	eBioscience	00-5523-00
*C. difficile* Toxin B (TcdB)	Abcam	ab124001
RNAlater	Invitrogen	AM7024
QuantiTect Reverse Transcription kit	Qiagen	205314
DyNamo ColorFlash Probe qPCR kit	Thermo Scientific	F456
QuantiNova SYBR Green PCR kit	Qiagen	208056
4 kDa fluorescein isothiocyanate (FITC) dextran	Sigma-Aldrich	46944
5-ethynyl-2′-deoxyuridine (EdU)	Invitrogen	A10044
Evans blue	Sigma-Aldrich	E2129

Critical commercial assays		

RNeasy mini kit	Qiagen	74106
DNeasy PowerSoil Pro kit	Qiagen	47016
Click-iT EdU Alexa Fluor 488 Flow Cytometry Kit	Invitrogen	C10425
Mouse Albumin ELISA kit	Bethyl Laboratories	E99-134
Urea assay kit	Abcam	ab83362
Creatinine assay kit	Abcam	ab65340
Glucose assay kit	Abcam	ab65333
Pierce ECL Western Blotting Substrate	Thermo Scientific	32109

Deposited data		

16S rRNA gene sequencing data	NCBI Bioproject https://www.ncbi.nlm.nih.gov/bioproject/	BioprojectID: PRJNA1133716
Flow cytometry data	Mendeley Data https://data.mendeley.com	https://doi.org/10.17632/npwvj89b6m.1
Experimental models: Cell lines		
Vero cells	ATCC	CCL-81
Caco-2 cells	ATCC	HTB-37

Experimental models: Organisms/strains		

C57BL/6 mice	Jackson Laboratory	000664
TLR-7 deficient mice	Jackson Laboratory	008380
IFNAR deficient mice	Jackson Laboratory	028288
Rag1 deficient mice	Jackson Laboratory	002216
IFN-γ deficient mice	Jackson Laboratory	002287
IL-22 deficient mice	Dr. Richard Flavell (Yale University)	N/A
Nfil3 deficient mice	Dr. Joseph Sun (Memorial Sloan Kettering Cancer Center)	N/A
Villin-Cre mice	Jackson Laboratory	004586
Stat3-flox mice	Jackson Laboratory	016923

Oligonucleotides		

TaqMan Gene Expression Assays, *Hprt*	Thermo Scientific	Mm03024075_m1
TaqMan Gene Expression Assays, *Reg3g*	Thermo Scientific	Mm00441127_m1
TaqMan Gene Expression Assays, *S100a8*	Thermo Scientific	Mm00496696_g1
TaqMan Gene Expression Assays, *Lcn2*	Thermo Scientific	Mm01324470_m1
TaqMan Gene Expression Assays, *Il22ra2*	Thermo Scientific	Mm01192969
QuantiTect Primer Assays, *Hprt*	Qiagen	QT00166768
QuantiTect Primer Assays, *Ifng*	Qiagen	QT01038821
QuantiTect Primer Assays, *Il23a*	Qiagen	QT01663613
QuantiTect Primer Assays, *Il22*	Qiagen	QT00128324
QuantiTect Primer Assays, *Cxcl1*	Qiagen	QT00115647
QuantiTect Primer Assays, *Ccl2*	Qiagen	QT00167832
QuantiTect Primer Assays, *Tnfa*	Qiagen	QT00104006
QuantiTect Primer Assays, *Il6*	Qiagen	QT00098875
QuantiTect Primer Assays, *Il1b*	Qiagen	QT01048355
QuantiTect Primer Assays, *Ifnb1*	Qiagen	QT00249662
QuantiTect Primer Assays, *Mgat4a*	Qiagen	QT00115892
QuantiTect Primer Assays, *Mgat4b*	Qiagen	QT01055593
QuantiTect Primer Assays, *Mgat5*	Qiagen	QT02523416
QuantiTect Primer Assays, *St6gal1*	Qiagen	QT02528400
QuantiTect Primer Assays, *Tjp1*	Qiagen	QT00493899
QuantiTect Primer Assays, *Ocln*	Qiagen	QT00111055
QuantiTect Primer Assays, *Cdh1*	Qiagen	QT00121163
QuantiTect Primer Assays, *Cldn2*	Qiagen	QT01059212

Software and algorithms		

GraphPad Prism (v.10.1.0)	GraphPad Software	N/A
NIS-Elements AR (v.5.20.01)	Nikon	N/A
ImageJ	National Institute of Health	N/A
FlowJo (v.10.10.0)	Becton Dickinson	N/A

## Highlights

- Oral administration of TLR7 agonist R848 protects mice against *C. difficile* infection
- R848-mediated protection is dependent on IL-22-producing ILC3s
- IL-22 signals through STAT3 on intestinal epithelial cells to facilitate mucosal repair
- R848 improves intestinal barrier integrity and limits systemic toxin dissemination
