# Supplementary material for: Therapeutic activation of IL-22-producing innate lymphoid cells enhances host defenses to Clostridioides difficile infection
Source: Cell Rep. Author manuscript; Available in PMC 2025 May 27. (PMC12115236; doi:10.1016/j.celrep.2025.115438)
Supplement: 1 [file NIHMS2076696-supplement-1.pdf]

Cell Reports, Volume *44*

Supplemental information

Therapeutic activation of IL-22-producing innate lymphoid cells enhances host defenses to *Clostridioides difficile* infection

Kevin S. Mears, Joshua E. Denny, Jeffrey R. Maslanka, Nontokozo V. Mdluli, Ellie N. Hulit, Rina Matsuda, Emma E. Furth, Charlie G. Buffie, and Michael C. Abt

Supplemental Information

Figure S1. R848 protects both male and female mice from *C. difficile* infection and reduces epithelial cell loss in the cecum. Related to Figure 1.

Antibiotics-treated C57BL/6 mice were treated with R848 and infected with *C. difficile.* Disease severity was assessed at day 2 p.i. (A) Males and females combined. (B) Separated by sex. Data combined from 4 experiments (n = 10 per group). (C) Histopathologic scoring of stained cecal sections from Figure 1I separated by category (cell infiltration, epithelial cell death, crypt hyperplasia, and intraluminal exudate) (n = 6-9 per group). Data is presented as mean ± SEM. Statistical significance was calculated by (A & C) unpaired t-test and (B) multiple t-test with two-way ANOVA with Holm-Šídák's correction. *p<0.05, **p<0.01.

Figure S2. Therapeutic R848 protects mice against severe disease, Related to Figure 1.

Antibiotic-treated C57BL/6 mice were treated with R848 from days -1 to 1 p.i. (full course), days -1 and 0 p.i. (prophylactic), or day 1 p.i. only (therapeutic). All mice received vehicle (H_2O) on days that they did not receive R848. Mice were infected with *C. difficile* and disease severity was assessed at day 2 p.i. (A) Schematic of experimental setup. (B) Day 2 disease severity. Data combined from 3 independent experiments (n = 15 per treatment group). Data is presented as mean ± SEM. Statistical significance was determined by one-way ANOVA with Tukey's correction. *p<0.05, **p<0.01.

Figure S3. R848 does not directly inhibit intestinal epithelial cell intoxication, Related to Figure 1.

Cultured intestinal epithelial cells (Caco-2) were treated with serial 10-fold dilutions of R848 (0.005-50 µg/mL) or media and intoxicated with purified TcdB (20 or 100 pM). Cell rounding was assessed as an indicator of intoxication. (A) Representative images of cells treated with 50 µg/mL of R848 or media (no R848) and 100 pM of TcdB or media (no TcdB). Images representative of 3 replicates. 50 µm scale bar. (B) Percent cell rounding at the various concentrations of R848 and TcdB tested. Cell rounding was estimated as complete (100%), incomplete but more than half (75%), approximately half (50%), or no rounding (0%). Data is presented as mean ± SEM.

Figure S4. R848 does not lead to changes in microbial composition, Related to Figure 1.

Fecal microbiota compositions were compared between treatment groups by 16S bacterial rRNA gene sequencing. (A) Principal coordinate analysis of weighted UniFrac distances between treatment groups at day 0 prior to *C. difficile* infection. (B) Relative abundances at the family level at day 0 with each bar representing an individual mouse.

Figure S5. Levels of inflammatory cell in the lamina propria are similar at 24 hours post-R848 treatment, Related to Figure 3.

(A) Representative gating for neutrophils and monocytes isolated from the large intestine lamina propria. Single cells, Live cells, $CD45^+$, non-T cells, non-B cells, $CD11b^+$, $Ly6c^+Ly6g^-$ (monocytes), $Ly6c^+Ly6g^+$ (neutrophils). C57BL/6 mice were treated with R848 or vehicle control and the (B) frequency and (C) total numbers of neutrophils and monocytes in the large intestine lamina propria were assessed at 24 hours by flow

cytometry. Data is presented as mean ± SEM. Statistical significance was determined by unpaired t test.

Figure S6. Flow cytometry gating for intracellular cytokine and intranuclear transcription factor expression, Related to Figure 3.

(A) Representative gating for intracellular cytokine staining of cells isolated from the colonic lamina propria. Single cells, Live cells, $CD45^+$, non-T cells, non-B cells, Gr-1^-, $CD90^+CD127^+$ (ILC). Single cells, Live cells, $CD45^+$, T cells, $\gamma\delta TCR^-CD4^+$ ($CD4^+$ T cells) and $\gamma\delta TCR^+CD4^-$ (γδ T cells). Cytokine gates set based on isotype negative control. (B) ILC transcription factor staining. Positive gates based on fluorescence-minus-one (FMO) staining (gray).

Figure S7. R848 does not stimulate IFN-γ and IL-22 production in $CD4^+$ T cells nor γδ T cells, Related to Figure 3.

C57BL/6 mice were treated with R848 or H_2O and T cell cytokine response was assessed 3 hours later in the large intestine lamina propria. (A) Frequency and (B) total number of IL-22 and IFN-γ producing $CD4^+$ T cells following ex vivo incubation in media with Brefeldin A (n = 5 per group). $CD4^+$ T cells were gated on live, $CD45^+$, $CD3/5^+$, $CD19^-$, $\gamma\delta TCR^-$, $CD4^+$ cells. (C) Frequency and (D) total number of IL-22 and IFN-γ producing γδ T cells (n = 5 per group). γδ T cells were gated on live, $CD45^+$, $CD3/5^+$, $CD19^-$, $CD4^-$, $\gamma\delta TCR^+$ cells. Data representative of two independent experiments. Data is presented as mean ± SEM. Statistical significance calculated by unpaired t-test.

Figure S8. R848 reduces colonic epithelial cell loss in an IL-22-dependent manner, Related to Figure 4.

Co-housed antibiotic-treated C57BL/6 and *Il22*$^{-/-}$ mice were orally administered R848 or H_2O and infected with *C. difficile*. (A) Representative images of H&E-stained sections of proximal colon day 2 p.i. 100 µm scale bar. (B) Histopathological scoring of stained proximal colon sections based on cell infiltration, epithelial cell death, crypt hyperplasia, and intraluminal exudate (n = 3-4 per group). (C) Histopathological scoring of epithelial cell death from (B). Data is presented as mean ± SEM. Statistical significance calculated by multiple t-test with Holm-Šídák’s correction.

Figure S9. The innate immune response at day 2 post-infection is comparable between R848 and H_2O treated mice, Related to Figure 4.

Antibiotic-treated C57BL/6 mice were orally administered R848 or H_2O and infected with *C. difficile*. (A) Expression of *Ifng, Il22, Tnfa, Il6, Il1b, Cxcl1, Ccl2, Reg3g, S100a8* and *Lcn2* mRNA transcripts at day 2 p.i. in whole proximal colon tissue as assessed by qRT-PCR and normalized to *Hprt* expression (n = 5 to 7). Data expressed as log2 fold-change relative to H_2O-treated uninfected mice. Data is a combination of two independent experiments. (B) Frequency and (C) total number of neutrophils and monocytes at day 2 p.i. (n = 5 to 13). FACS plots were gated on live, CD45^{+}, CD3^{-} CD5^{-}, γδTCR^{-}, NK1.1^{-}, CD19^{-}, Siglec-F^{-}, CD11b^{+} cells. Data combined from four experiments. (D) Frequency and (E) total number of ILCs isolated from the large intestine lamina propria (Lp) at day 2 p.i. producing IFN-γ or IL-22 following ex vivo incubation in media with Brefeldin A. (F) Frequency and (G) total number of ILCs isolated from the mesenteric lymph nodes (mLN)

at day 2 p.i. producing IFN-γ or IL-22 following ex vivo incubation in media with Brefeldin A. FACS plots gated on live, $CD45^+$, $CD3^-$, $CD5^-$, $CD8\alpha^-$, $CD19^-$, Gr-1^-, $CD90^+CD127^+$ cells. Data combined from two independent experiments. Data is presented as mean ± SEM. Statistical significance was calculated by unpaired t-test with Holm-Šídák's multiple comparison correction. *p<0.05, **p<0.01, ***p<0.001.

Figure S10. Expression of select epithelial glycosylation and junction genes is decreased by R848 treatment in an IL-22-dependent manner, Related to Figure 5.

Co-housed C57BL/6 and *Il22*-/- mice were treated with R848 or H_2O and large intestine epithelial cells were collected at 3 or 24 hours later. Expression of (A) glycosyltransferase genes *Mgat4a, Mgat4b, Mgat5, St6gal1,* and (B) junction genes *Tjp1, Ocln, Cdh1, Cldn2* were assessed by qRT-PCR. Data normalized to *Hprt* expression and expressed as log2 fold-change relative to C57BL/6 H_2O-treated controls (n = 5 per group). Data is presented as mean ± SEM. Statistical significance was calculated by 2-way ANOVA with Dunnett's correction. *p<0.05, **p<0.01.

Figure S11. Flow cytometry gating for EdU incorporation in intestinal stem cells, Related to Figure 5.

(A) Representative gating for EdU^+ intestinal stem cells. Single cells were gated on live, $Epcam^+$, $CD45^{neg}$, $CD44^{hi}$, $CD24^{lo}$, c-Kit^{neg} cells. EdU gate was set according to mice that did not receive EdU injection.

Figure S12. R848 does not reduce vascular permeability at day 2 post-infection, Related to Figure 5.

Antibiotic-treated C57BL/6 mice were orally administered R848 or H_2O and infected with *C. difficile*. At day 2 p.i., mice were retro-orbitally injected with Evans blue dye to assess intestinal and systemic vascular permeability. Concentration of Evans blue in (A) kidney and (B) cecal tissue. Data is presented as mean ± SEM. Statistical significance was calculated by two-way ANOVA with Šídák’s correction. *$p<0.05$.

Figure S1

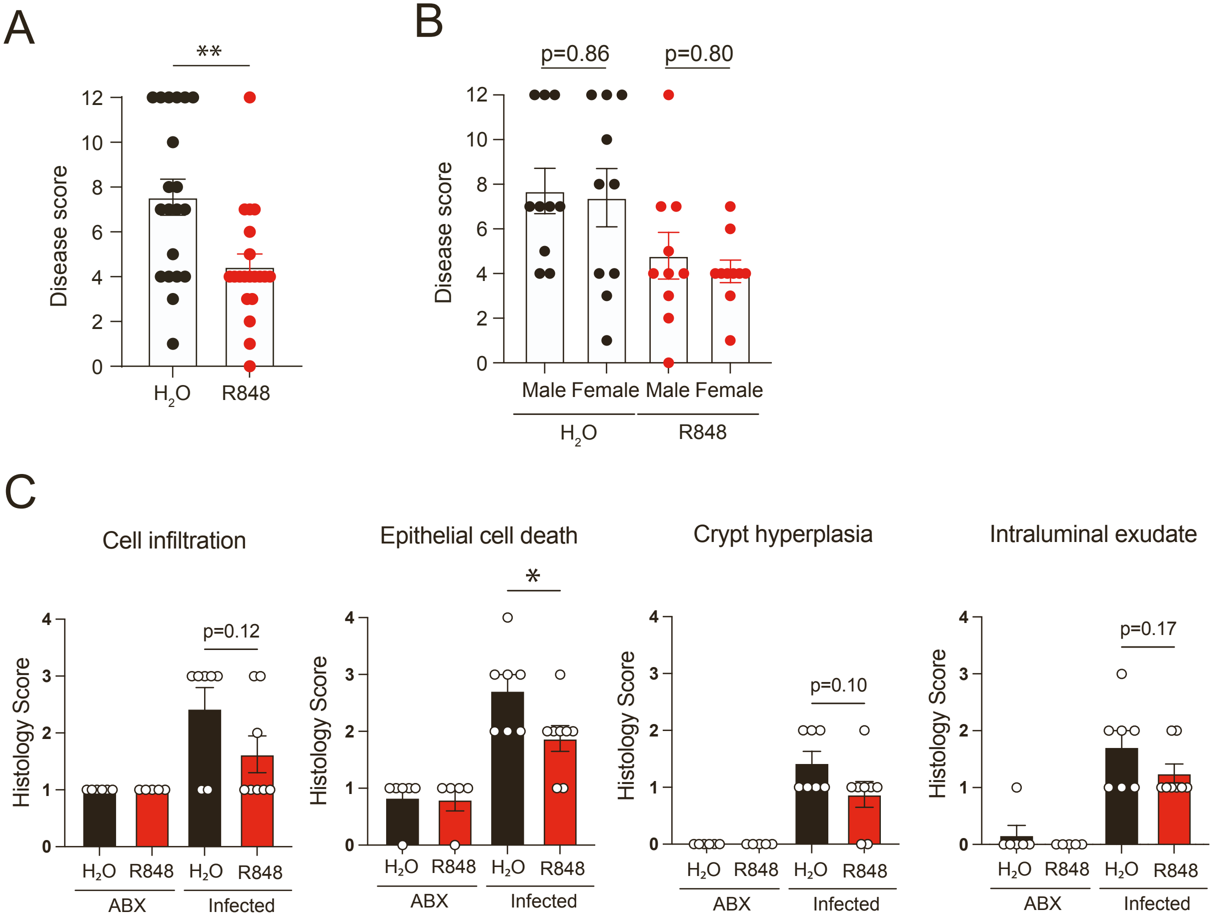
A
**
12
10
8
6
4
2
0
Disease score
H2O
R848
B
p=0.86
p=0.80
12
10
8
6
4
2
0
Disease score
Male Female Male Female
H2O
R848
C
Cell infiltration
Epithelial cell death
Crypt hyperplasia
Intraluminal exudate
p=0.12
*
p=0.10
p=0.17
4
3
2
1
0
Histology Score
H2O R848 H2O R848
ABX
Infected

Figure S2

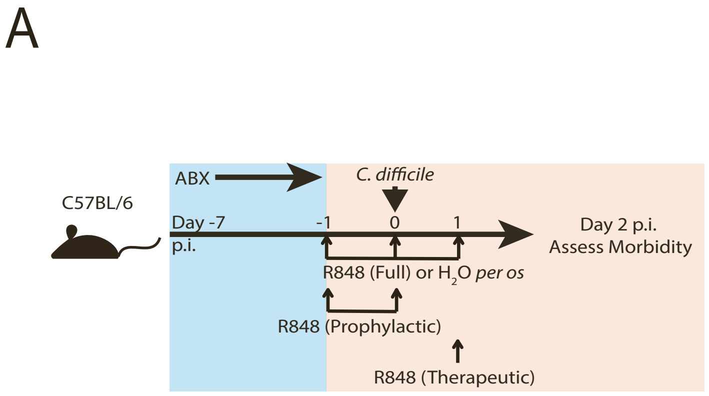
A
C57BL/6
ABX
C. difficile
Day -7
p.i.
-1
0
1
Day 2 p.i.
Assess Morbidity
R848 (Full) or H2O per os
R848 (Prophylactic)
R848 (Therapeutic)

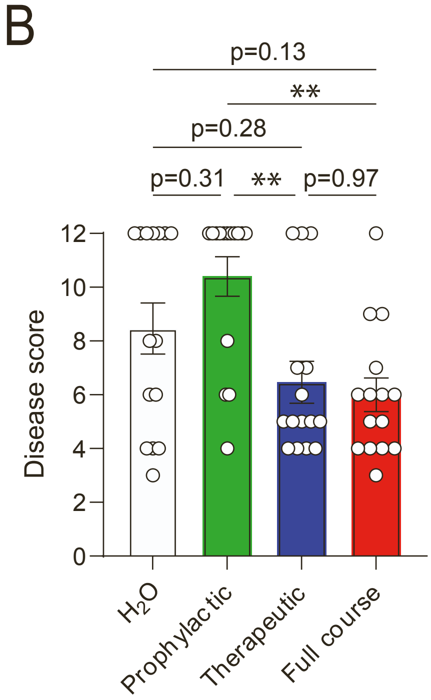
B
p=0.13
**
p=0.28
p=0.31
**
p=0.97
Disease score
12
10
8
6
4
2
0
H2O
Prophylactic
Therapeutic
Full course

Figure S3

A

No R848 | R848 (50 μg/mL)

No TcdB

TcdB (100pM)

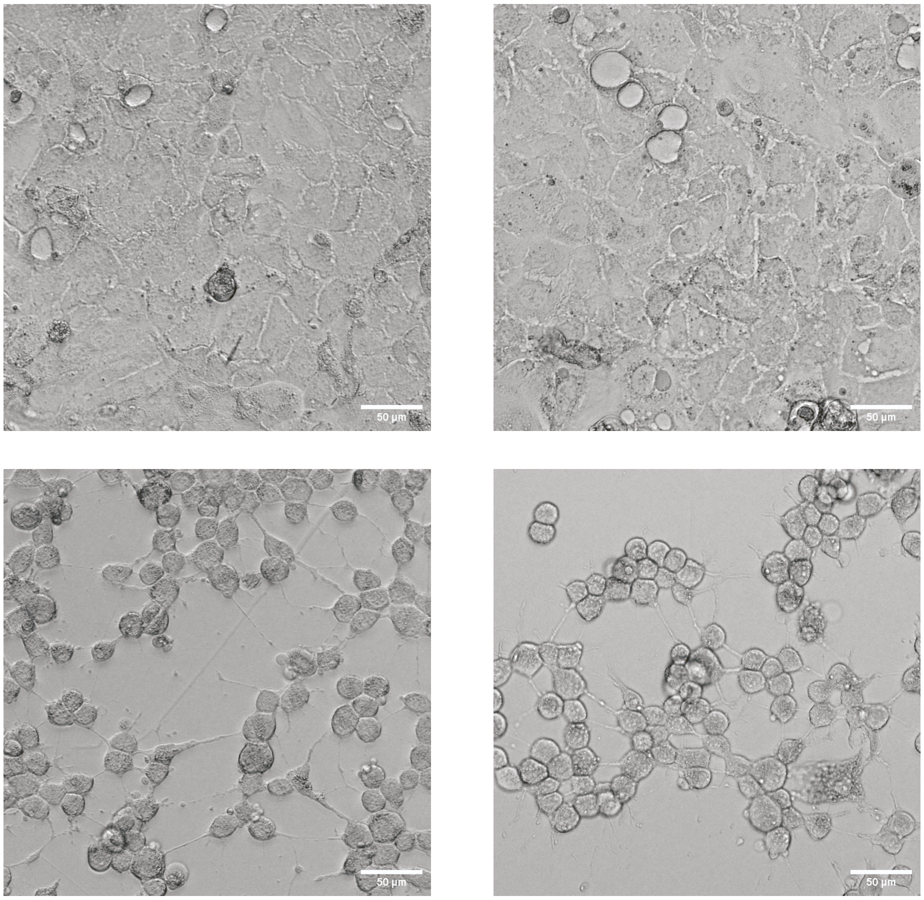

B

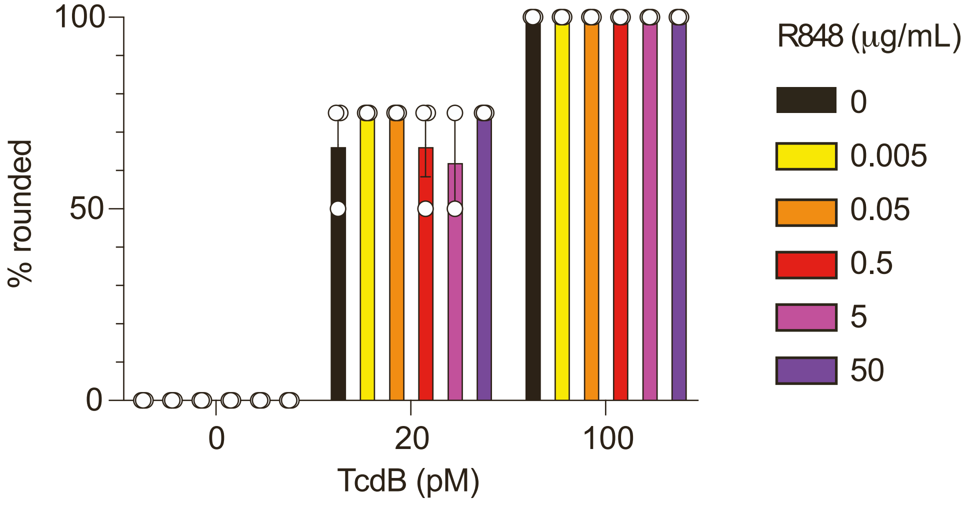

Figure S4

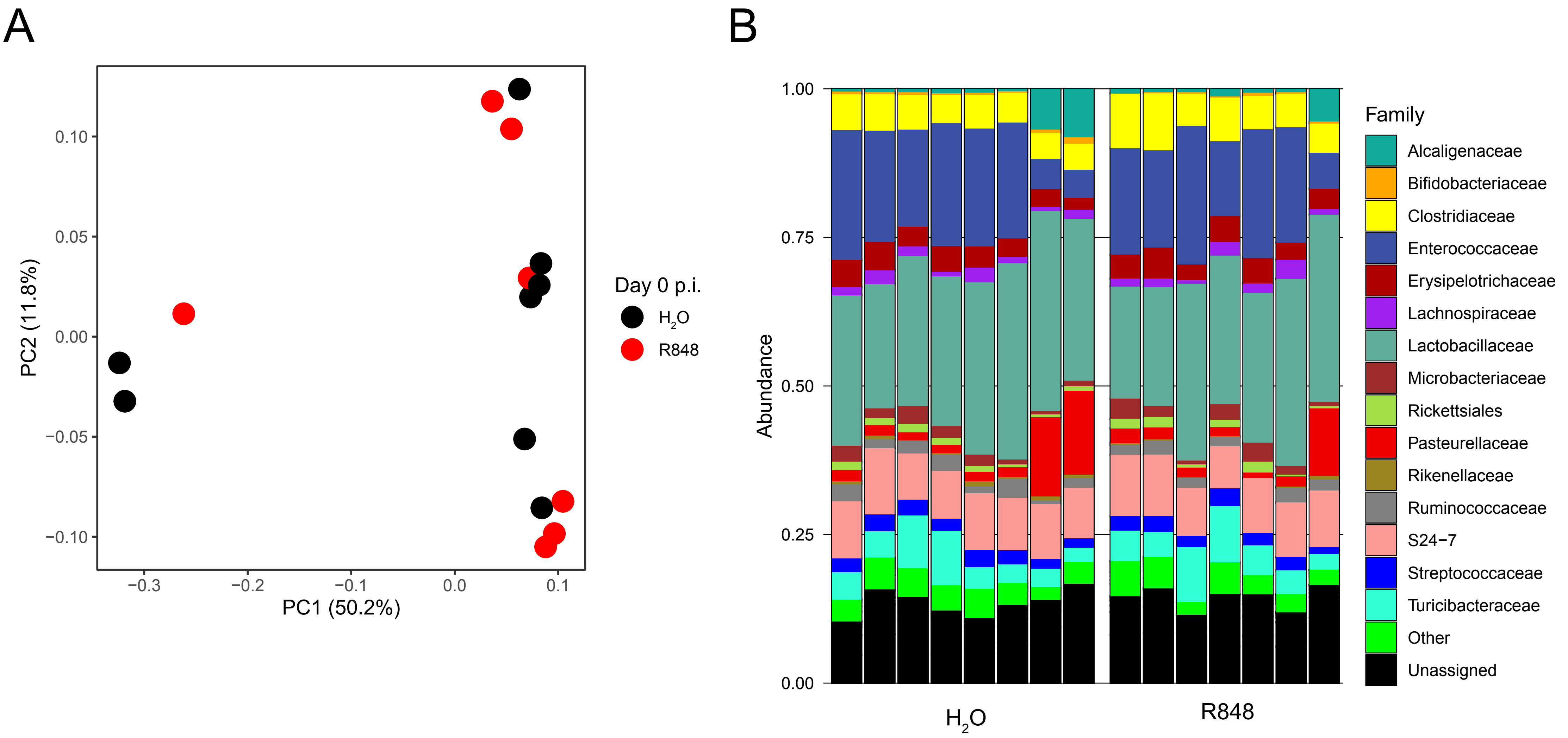

A
B
PC1 (50.2%)
PC2 (11.8%)
Day 0 p.i.
H2O
R848
Abundance
Family
Alcaligenaceae
Bifidobacteriaceae
Clostridiaceae
Enterococcaceae
Erysipelotrichaceae
Lachnospiraceae
Lactobacillaceae
Microbacteriaceae
Rickettsiales
Pasteurellaceae
Rikenellaceae
Ruminococcaceae
S24−7
Streptococcaceae
Turicibacteraceae
Other
Unassigned

Figure S5

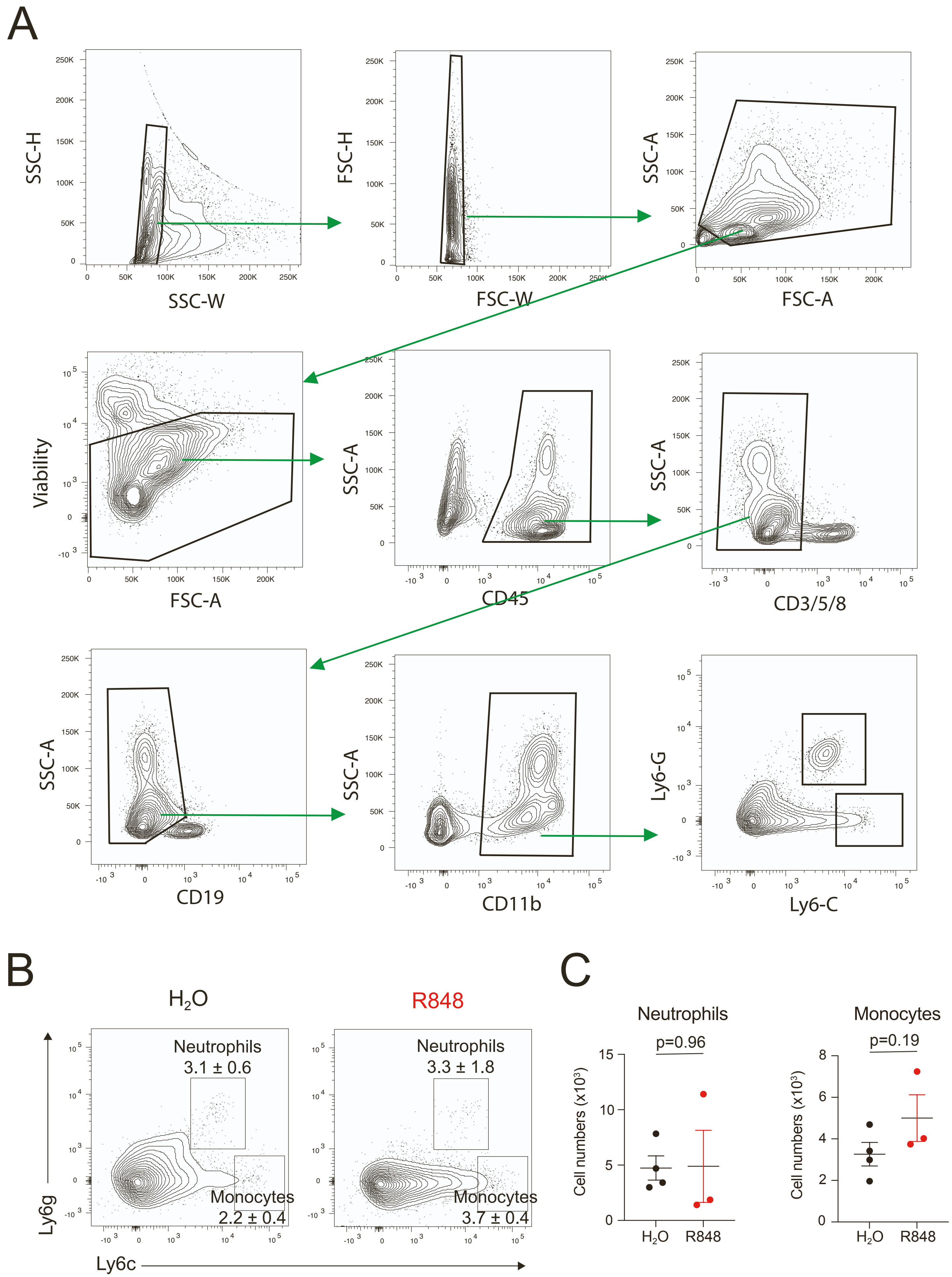
A
SSC-H
SSC-W
FSC-H
FSC-W
SSC-A
FSC-A
Viability
CD45
CD3/5/8
CD19
CD11b
Ly6-G
Ly6-C
B
H_2O
R848
Neutrophils
3.1 ± 0.6
Monocytes
2.2 ± 0.4
Neutrophils
3.3 ± 1.8
Monocytes
3.7 ± 0.4
Ly6g
Ly6c
C
Neutrophils
p=0.96
Monocytes
p=0.19
Cell numbers ($x10^3$)
H_2O
R848

Figure S6

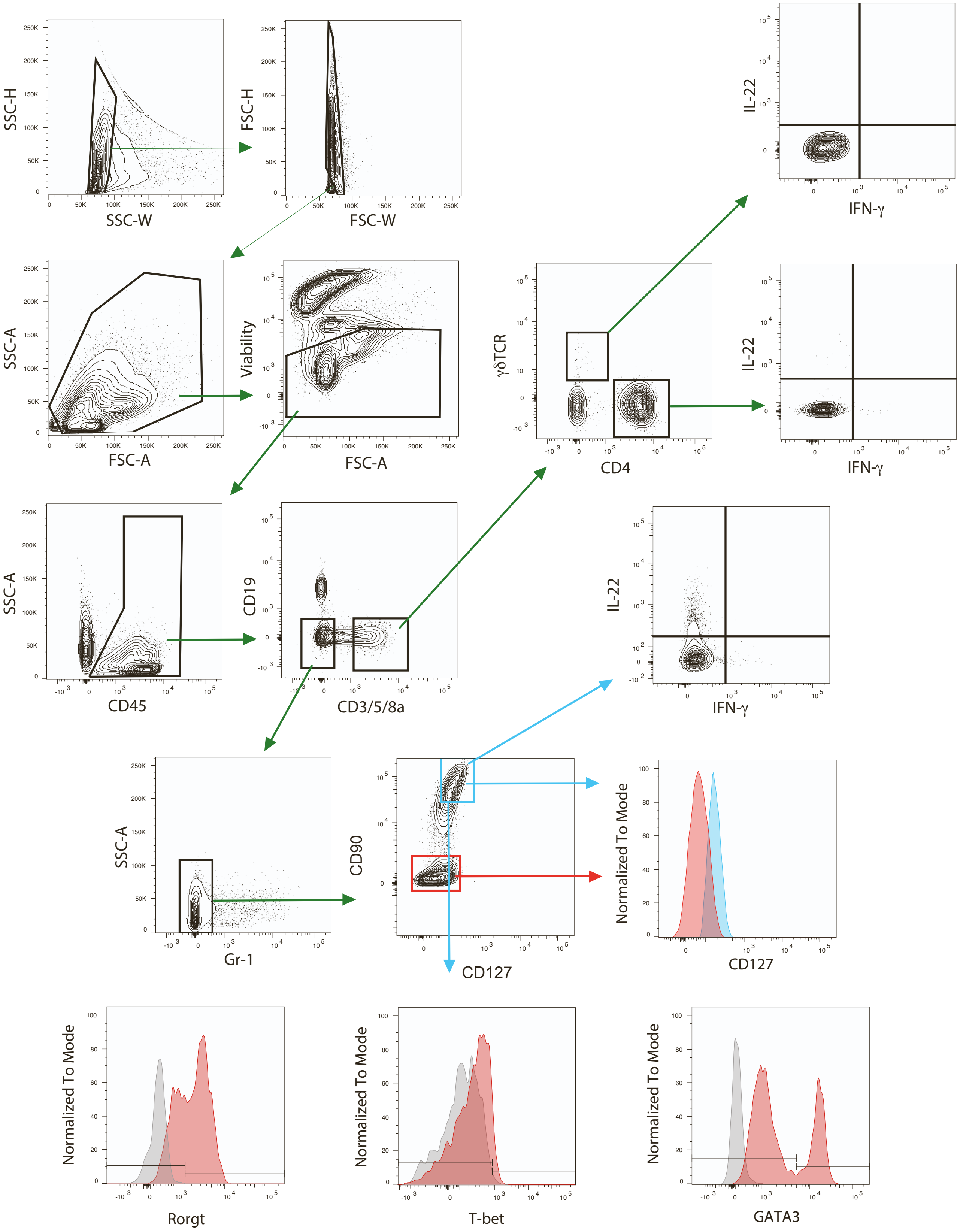

SSC-H
SSC-W
FSC-H
FSC-W
IL-22
IFN-γ
SSC-A
FSC-A
Viability
γδTCR
CD4
CD45
CD19
CD3/5/8a
Gr-1
CD90
CD127
Normalized To Mode
Rorgt
T-bet
GATA3

Figure S7

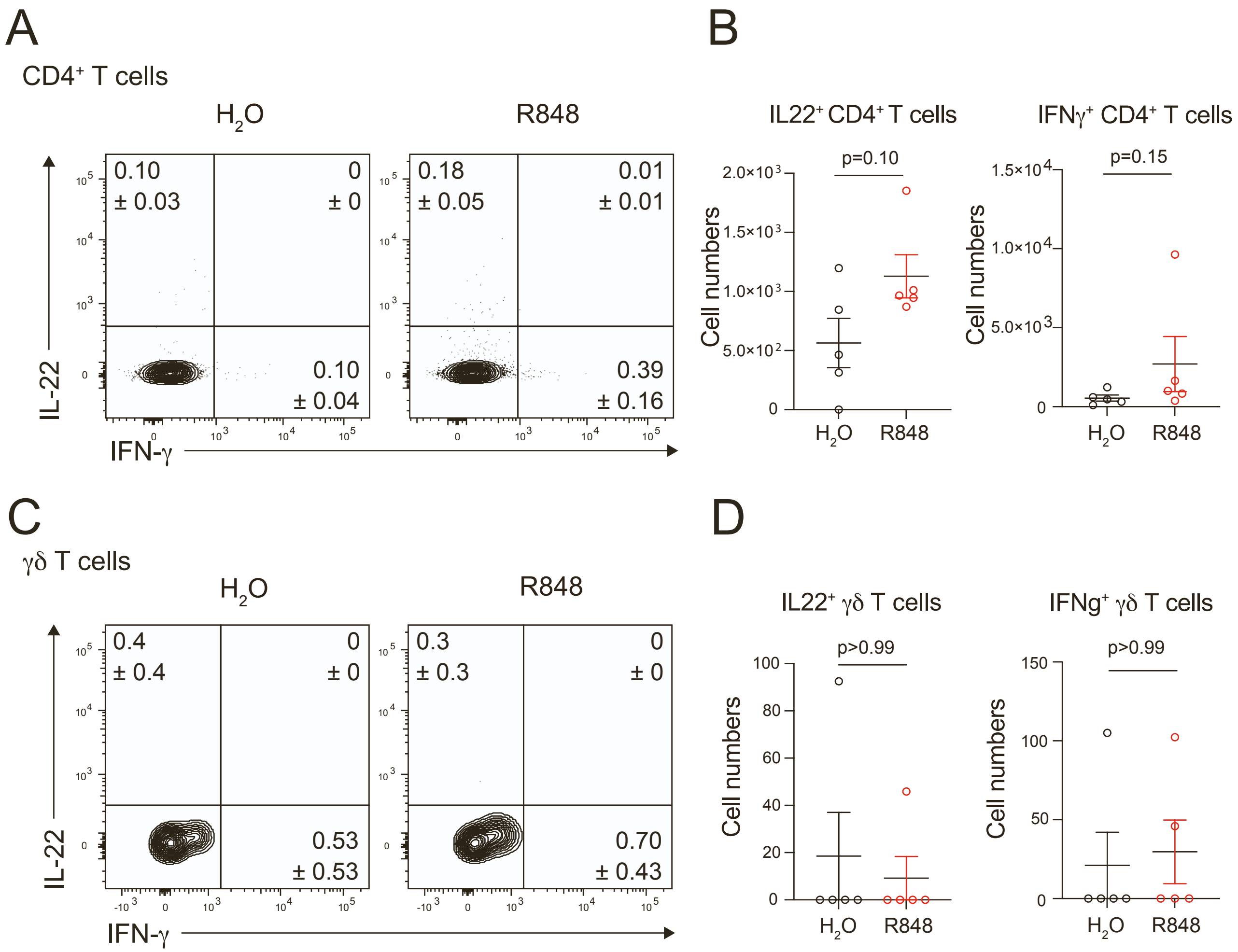
A
CD4+ T cells
H2O
R848
0.10 ± 0.03
0 ± 0
0.10 ± 0.04
0.18 ± 0.05
0.01 ± 0.01
0.39 ± 0.16
IL-22
IFN-γ
B
IL22+ CD4+ T cells
IFNγ+ CD4+ T cells
p=0.10
p=0.15
Cell numbers
H2O
R848
C
γδ T cells
H2O
R848
0.4 ± 0.4
0 ± 0
0.53 ± 0.53
0.3 ± 0.3
0 ± 0
0.70 ± 0.43
IL-22
IFN-γ
D
IL22+ γδ T cells
IFNg+ γδ T cells
p>0.99
p>0.99
Cell numbers
H2O
R848

Figure S8

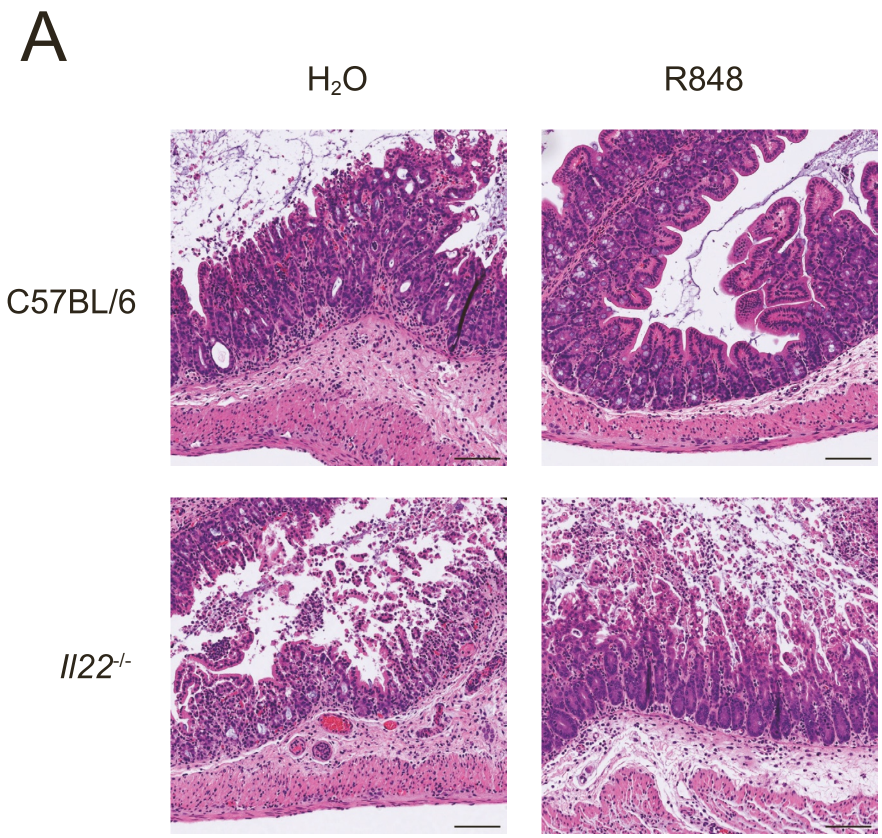
A
H2O
R848
C57BL/6
Il22-/-

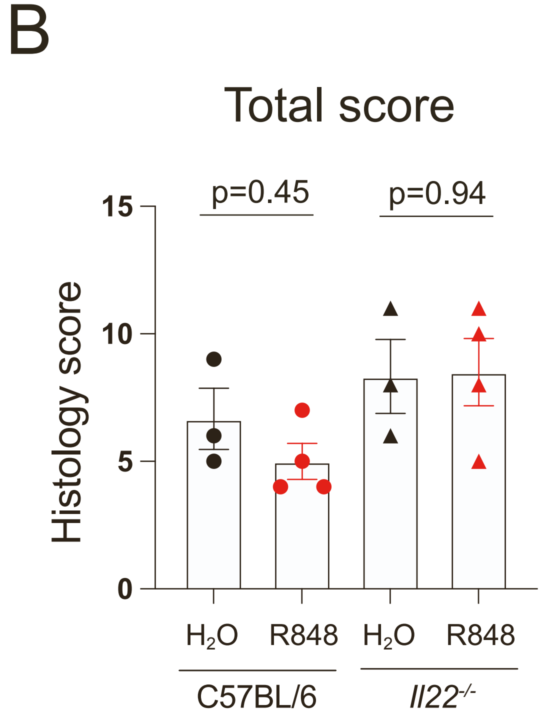
B
Total score
Histology score
15
10
5
0
p=0.45
p=0.94
H2O
R848
H2O
R848
C57BL/6
Il22-/-

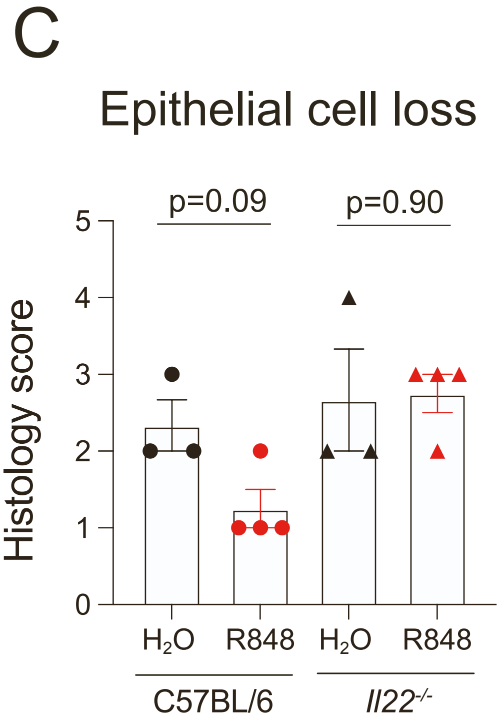
C
Epithelial cell loss
Histology score
5
4
3
2
1
0
p=0.09
p=0.90
H2O
R848
H2O
R848
C57BL/6
Il22-/-

Figure S9

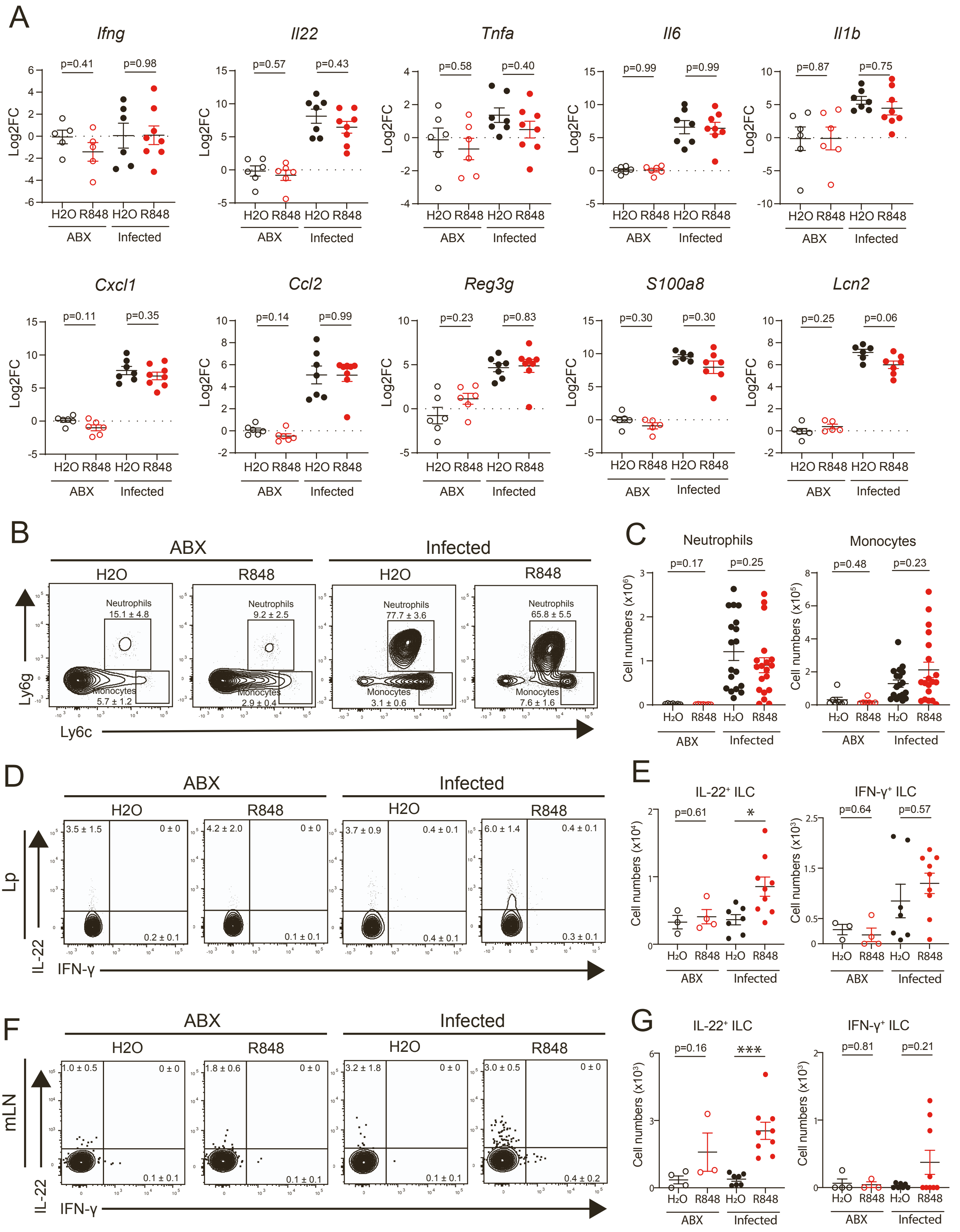
A
Ifng
Il22
Tnfa
Il6
Il1b
Cxcl1
Ccl2
Reg3g
S100a8
Lcn2
Log2FC
H2O R848 H2O R848
ABX
Infected
p=0.41
p=0.98
p=0.57
p=0.43
p=0.58
p=0.40
p=0.99
p=0.99
p=0.87
p=0.75
p=0.11
p=0.35
p=0.14
p=0.99
p=0.23
p=0.83
p=0.30
p=0.30
p=0.25
p=0.06
B
ABX
Infected
H2O
R848
Neutrophils
15.1 ± 4.8
9.2 ± 2.5
77.7 ± 3.6
65.8 ± 5.5
Monocytes
5.7 ± 1.2
2.9 ± 0.4
3.1 ± 0.6
7.6 ± 1.6
Ly6g
Ly6c
C
Neutrophils
Monocytes
Cell numbers (x10^6)
Cell numbers (x10^5)
p=0.17
p=0.25
p=0.48
p=0.23
D
Lp
IL-22
IFN-γ
3.5 ± 1.5
0 ± 0
0.2 ± 0.1
4.2 ± 2.0
0 ± 0
0.1 ± 0.1
3.7 ± 0.9
0.4 ± 0.1
0.4 ± 0.1
6.0 ± 1.4
0.4 ± 0.1
0.3 ± 0.1
E
IL-22+ ILC
IFN-γ+ ILC
Cell numbers (x10^4)
Cell numbers (x10^3)
p=0.61
*
p=0.64
p=0.57
F
mLN
1.0 ± 0.5
0 ± 0
0.1 ± 0.1
1.8 ± 0.6
0 ± 0
0.1 ± 0.1
3.2 ± 1.8
0 ± 0
0.1 ± 0.1
3.0 ± 0.5
0 ± 0
0.4 ± 0.2
G
IL-22+ ILC
IFN-γ+ ILC
Cell numbers (x10^3)
p=0.16
***
p=0.81
p=0.21

Figure S10

A

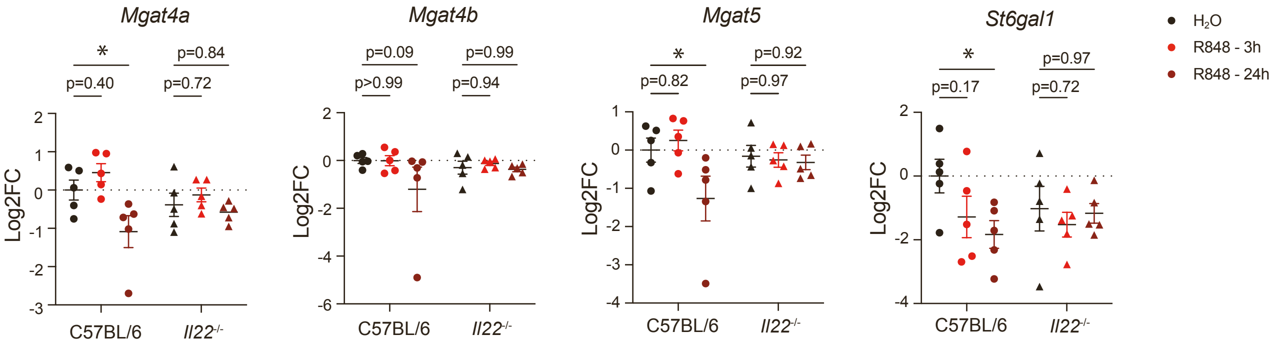

B

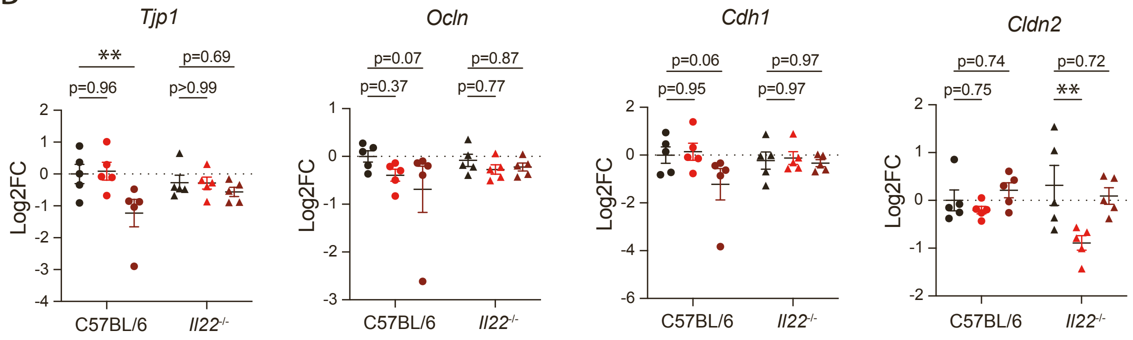

Figure S11

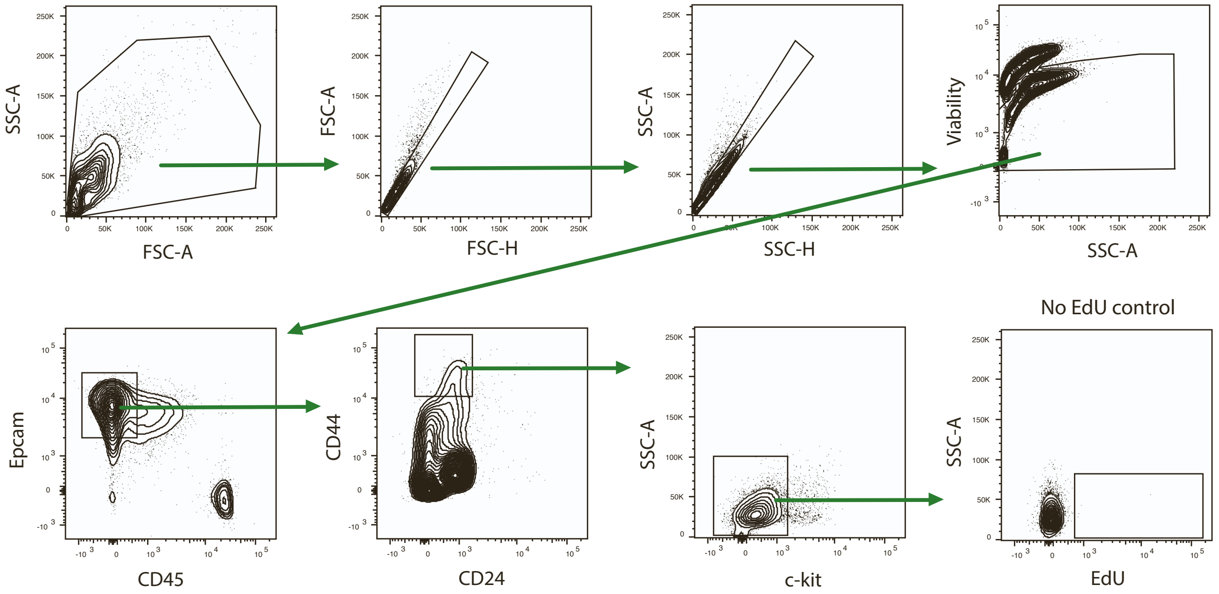
SSC-A
FSC-A
FSC-A
FSC-H
SSC-A
SSC-H
Viability
SSC-A
Epcam
CD45
CD44
CD24
SSC-A
c-kit
No EdU control
SSC-A
EdU

Figure S12

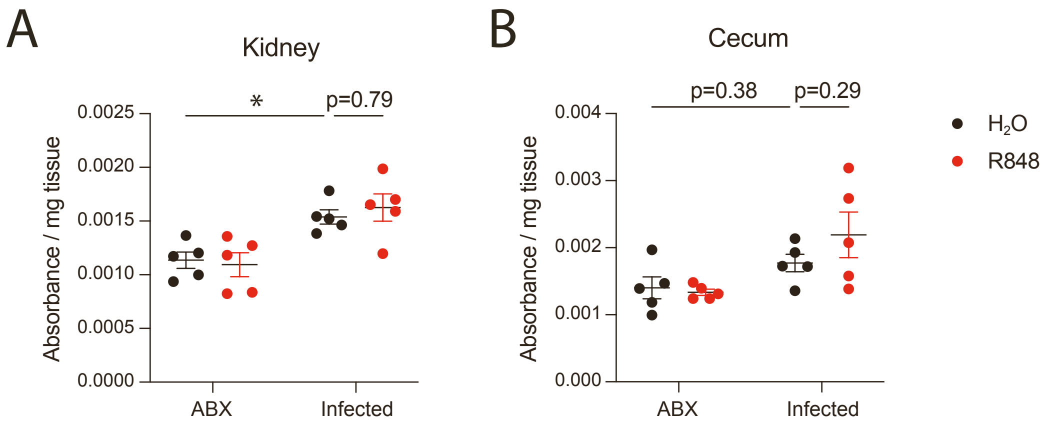

A
Kidney
*
p=0.79
Absorbance / mg tissue
0.0025
0.0020
0.0015
0.0010
0.0005
0.0000
ABX
Infected
B
Cecum
p=0.38
p=0.29
Absorbance / mg tissue
0.004
0.003
0.002
0.001
0.000
ABX
Infected
H_2O
R848
